# Tubule-specific protein nanocages potentiate targeted renal fibrosis therapy

**DOI:** 10.1186/s12951-021-00900-w

**Published:** 2021-05-26

**Authors:** Xuan Zhang, Qian Chen, Liyuan Zhang, Haiping Zheng, Chunjie Lin, Qunfang Yang, Tao Liu, Haigang Zhang, Xiaohong Chen, Lei Ren, Wenjun Shan

**Affiliations:** 1 Department of Pharmacology, College of Pharmacy and Laboratory Medicine, Army Medical University (Third Military Medical University), Chongqing, 400038 People’s Republic of China; 2 Biomedical Analysis Center, College of Basic Medicine, Army Medical University (Third Military Medical University), Chongqing, 400038 People’s Republic of China; 3 School of Medicine, Xiamen University, Xiamen, 361102 People’s Republic of China; 4 School of Life Sciences, Xiamen University, Xiamen, 361102 People’s Republic of China; 5 Department of Biomaterials, College of Materials, Xiamen University, Xiamen, 361005 People’s Republic of China; 6 School of Medicine, Huaqiao University, Quanzhou, 362021 People’s Republic of China

**Keywords:** Protein nanocage, Celastrol, Target therapy, Cell senescence, Renal fibrosis

## Abstract

**Background:**

Despite the dramatic advances in modern medicine, efficient therapeutic measures for renal fibrosis remain limited. Celastrol (CLT) is effective in treating renal fibrosis in rat models, while causing severe systemic toxicity. Thus, we designed a tubule-specific nanocage (K3-HBc NCs) that effectively deliver CLT to tubular epithelial cell in a virus-like manner. The targeting ligand (K3) to tubular epithelial cells was displayed on the surface of Hepatitis B core protein (HBc) NCs by genetic fusion to the major immunodominant loop region. Ultra-small CLT nanodots were subtly encapsulated into the cavity through electrostatic interaction with the disassembly and reassembly of K3-HBc NCs, to yield K3-HBc/CLT complex. The efficacy of K3-HBc/CLT NCs were demonstrated in Unilateral ureteral obstruction (UUO)-induced renal fibrosis.

**Results:**

The self-assembled K3-HBc/CLT could specifically target tubular epithelial cells via affinity with K3 ligand binding to the megalin receptor, significantly attenuating renal fibrosis. Remarkably, K3-HBc/CLT NCs significantly increased therapeutic efficacy and reduced the systemic toxicity in comparison with free CLT in UUO-induced mouse renal fibrosis model. Importantly, analysis of RNA sequencing data suggested that the anti-fibrotic effect of K3-HBc/CLT could be attributed to suppression of premature senescence in tubular epithelial cells via p21^Cip1^ and p16^Ink4a^ pathway.

**Conclusion:**

The tubule-specific K3-HBc/CLT represented a promising option to realize precise treatment for renal fibrosis.

**Supplementary Information:**

The online version contains supplementary material available at 10.1186/s12951-021-00900-w.

## Introduction

Chronic kidney disease (CKD) is a major public health concern worldwide [1]. Renal fibrosis is the common ultimate outcome of CKD and leads to irreversible loss of renal parenchyma and end-stage renal failure where the patients require dialysis or renal transplantation [2]. So far, fibrotic nephropathy is a severe clinical problem without effective treatment strategies and its pathogenesis remains to be elucidated [3, 4].

For a long time, many therapeutic approaches have focused on fibroblasts and myofibroblasts as these cells are the effectors of renal fibrosis [5]. However, these treatments have been disappointing. A number of important recent advances have clarified that the injured tubular epithelial cell is an important orchestrator in the process of renal fibrosis [6]. Tubular epithelial cells are essential for injury. They can adopt different repair mechanisms to survive injury and recover normal kidney function. But as the injury is severe and persistent, normal regeneration mechanisms may not work, and tubular epithelial cells might undergo maladaptive repair, which promote tubular atrophy and exacerbate renal fibrosis. The maladaptive tubular epithelial cells can be transformed into a secreted phenotype, thereby generating and releasing various biologically active molecules to precipitate the recruitment of inflammatory cells, the activation of fibroblasts and the loss of endothelial cells, which ultimately drives tubulointerstitial inflammation and fibrosis [7]. Furthermore, tubular epithelial cells can directly contribute to renal fibrosis via epithelial–mesenchymal transition (EMT), a phenotypic conversion program [8]. Tubular epithelial cells could be a promising novel target for ameliorating or even reversing renal fibrosis, but have received considerably less attention.

Natural products play a critical role in finding potential therapeutic agents [9]. For example, Tripterygiumwilfordii Hook F (TWHF), a traditional Chinese medicine, has been widely used in CKD in China for a long time [10], due to its strong anti-inflammatory and immunosuppressive effects [11]. Celastrol (CLT) is one of the predominantly active products in TWHF formulations and has the potential to protect kidney structure and function during chronic kidney injury. However, CLT was reported to induce severe cardiotoxicity, hepatotoxicity, neurotoxicity and reproductive disorder after systemic administration [12]. Meanwhile, poor water solubility (13.25 ± 0.83 μg mL^−1^ at 37 °C), low bioavailability and narrow window of dosage also restricted its further therapeutic application [13]. According to these rationales, targeted delivery of potentially therapeutic CLT to tubular epithelial cells could be an effective antifibrotic strategy.

Natural nanoparticles such as viruses possess their own delivery mechanisms that bear a striking resemblance to the action of many drug delivery vectors [14]. They can cross the human biological barrier easily through specific interactions with target cells. Protein nanocages (NCs) that mimic the overall structure and features of authentic virus without containing viral genetic materials are attractive drug delivery platforms [15]. They are formed by the natural self-assembly of protein subunits, and their repetitive surfaces can be bioengineered by specific ligand to recognize target cell [16]. Their cavity inside the particles can be used to encapsulate therapeutic agents. Moreover, chemical and genetic modifications of NCs can converge the advantageous properties to improve the therapeutic efficiencies.

**Scheme 1:**
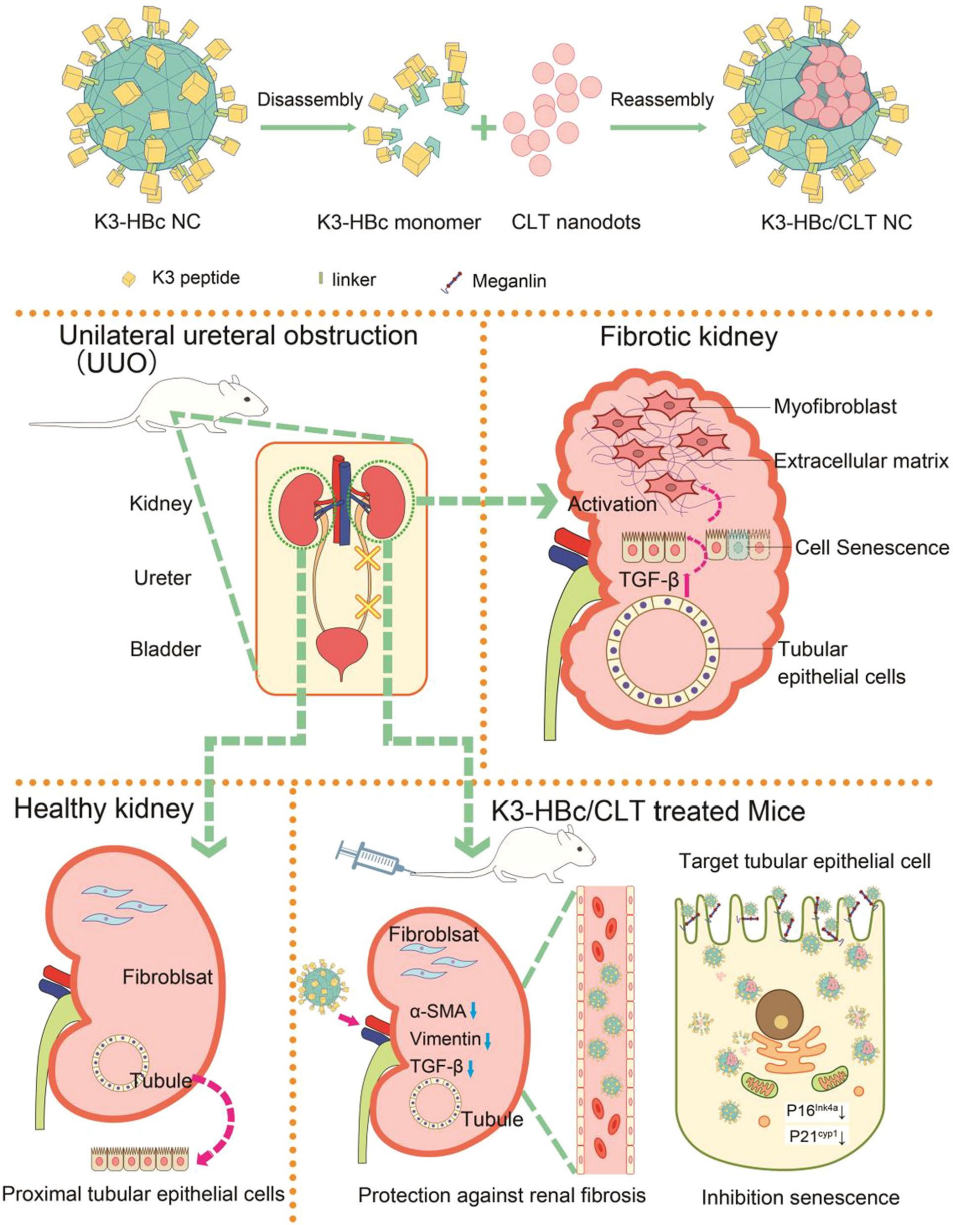
Schematic of tubule-specific delivery of CLT using virus-mimetic protein nanocages in UUO-induced renal fibrosis

Herein, we designed a biomimetic nanosystem that could precisely deliver CLT to tubular epithelial cells via a virus-like mechanism. We prepared the bioengineered Hepatitis B core protein **(**HBc) NCs to specifically recognize tubular epithelial cells. The peptide sequence of (KKEEE)3 (K3), a targeting ligand to tubular epithelial cells [17], was exposed on the surface of the nanocage (K3-HBc NCs). Ultra-small CLT nanodots were subtly encapsulated into the cavity through electrostatic interaction with the disassembly and reassembly of K3-HBc NCs, to yield K3-HBc/CLT complex. The resultant particles thus gain a uniform structure, controlled self-assembly, well biocompatibility and biodegradability, as well as high specificity. Therefore, the present complex achieved high-efficient targeted therapy for renal fibrosis (Scheme 1).

## Results

### CLT attenuates unilateral ureteral obstruction (UUO)-induced renal fibrosis with severe toxicity

We first studied the therapeutic effect of CLT. In the UUO group, CLT (1 mg kg^−1^) was administered by intraperitoneal injection every other day. The ratio of kidney weight to body weight (kidney index) after 2 weeks treatment was shown in Additional file 1: Figure S1A. The left kidney index was 4.23 ± 0.05% in sham group, whereas it increased to 6.19 ± 0.86% in UUO + 0.9% NaCl group. However, treatment with CLT reduced the left kidney index to 5.91 ± 0.39%. Since all the right kidney in each group was not obstructed, there was no significant change in the right kidney index. Increased kidney index implied loss of kidney function, treatment with CLT might rescue some of the negative impact of UUO on kidney function. The hematoxylin and eosin (H&E) staining revealed that CLT-treated rats had less fibrotic changes in the obstructed kidney than 0.9%-NaCl-treated rats (Additional file 1: Figure S1B). Renal fibrosis was much attributed to excess deposition of extracellular matrix components such as collagen [2]. We then examined the collagen deposition in renal tissue using Masson‘s trichrome staining. While the collagen deposition increased significantly in the rats subjected to UUO, a marked reduction was detected in CLT-treated rats (Additional file 1: Figure S1C). The fibrotic area represented by Masson’s trichrome staining in each group was analyzed in Additional file 1: Figure S1D. The fibrotic area value was 5.54 ± 0.82 in sham group, compared with a robust elevation to 183.29 ± 7.98 in UUO + 0.9% NaCl group. We found that CLT could decrease this value to 17.06 ± 3.22, implying that more than 90% collagen was eliminated. α-smooth muscle actin (α-SMA), a major morphological characteristic of myofibroblasts, plays a crucial role in the development and progression of renal tubulointerstitial fibrosis [18]. In Additional file 1: Figure S1E and F, the extremely high expression level of α-SMA in UUO + 0.9% NaCl group was decreased from 154.98 ± 34.10 (fold of sham group) to 41.16 ± 8.39 by CLT. The immunostaining of α-SMA in the renal tissue, as well as another two important fibrotic markers including transforming growth factor-beta (TGF-β) and collagen I, was further carried out. Similarly, elevated α-SMA expression (Additional file 1: Figure S1G and H) in the UUO group and its decrease in response to CLT was confirmed. Among a panel of fibrogenic factors, TGF-β is considered a key regulatory molecule in renal fibrotic process [19]. Although TGF-β was expressed at highly elevated levels in the renal tubular epithelial cells circulating around the tubules in UUO + 0.9% NaCl group (Additional file 1: Figure S1G), the integrated optical density (IOD) of TGF-β was decreased from 55.46 ± 6.29 to 15.94 ± 3.22 by CLT (Additional file 1: Figure S1I). The collagen Ι expressions was significantly lower in UUO + CLT group than in UUO + 0.9% NaCl group (Additional file 1: Figure S1G, J). These findings suggested that CLT harbored a potential therapeutic effect on renal fibrosis. However, the systemic toxic effects caused by CLT were inevitable. The rats with free CLT had severe weight loss, while the rats in other groups appeared normal (Additional file 1: Figure S1K). Moreover, serious perivisceral adhesion was found in the healthy rats after administration of free CLT. The toxic effect of CLT on major organs such as heart, liver, spleen, lung, and brain were further examined by H&E staining (Additional file 1: Figure S2). Especially, myofibrillar damage, cellular atrophy in heart samples, dilatation of blood sinus in the liver and pyknosis of neurons in brain were observed. Taking together, CLT showed extraordinary protective effect against renal fibrosis while accompanied by severe toxicity effects.

### Characterization of K3-HBc/CLT NCs

**Fig. 1:**
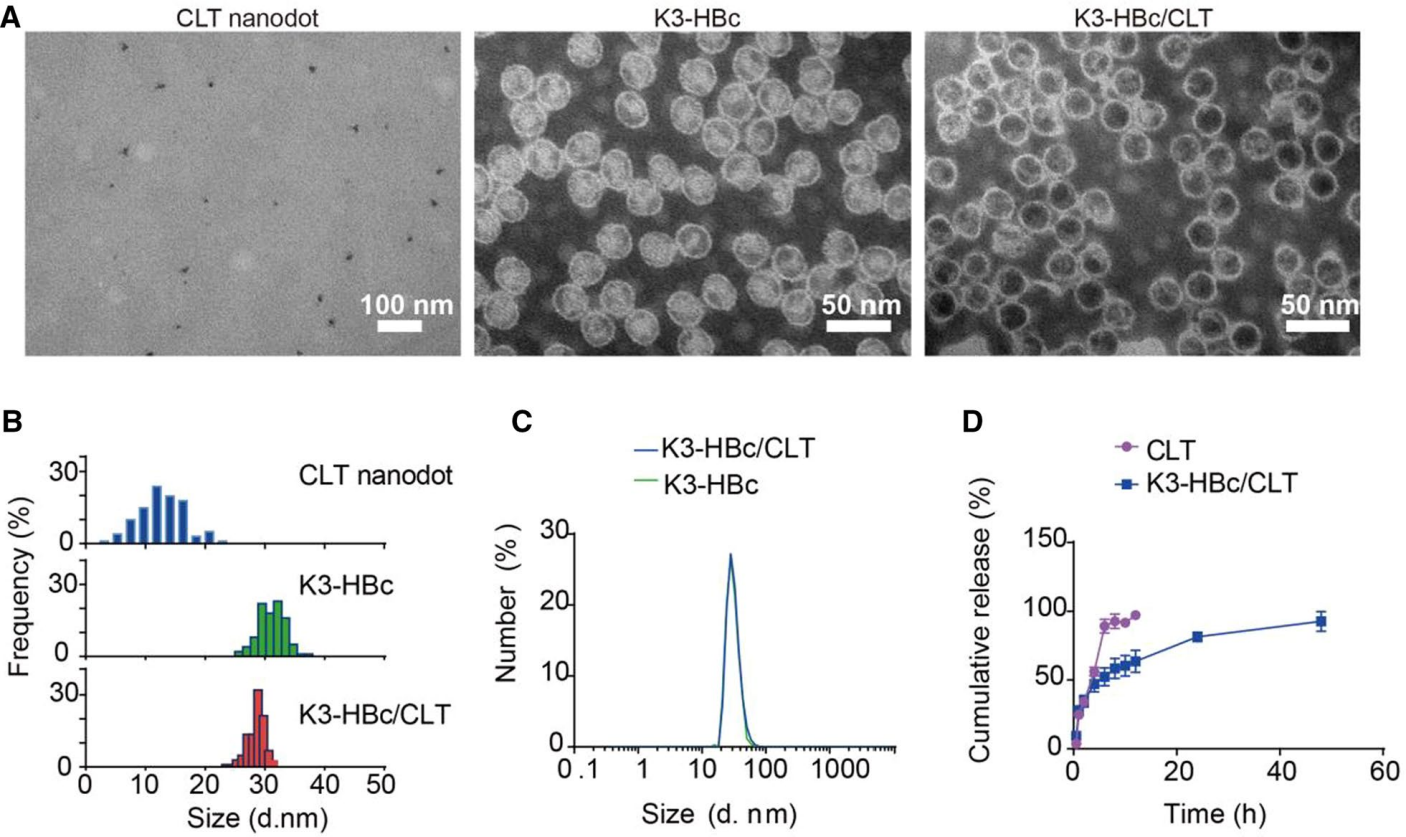
Characterization of K3-HBc/CLT NCs. **A** TEM images of CLT nanodots, K3-HBc NCs and K3-HBc/CLT NCs. **B** Histogram analysis of CLT nanodots, K3-HBc NCs and K3-HBc/CLT NCs, respectively. **C** The hydrodynamic diameters of K3-HBc and K3-HBc/CLT NCs. **D** Cumulative release profiles of free CLT and K3-HBc/CLT (n = 3)

We next tried to develop virus-mimetic protein nanoparticles derived from HBc NCs for targeting delivery of CLT. HBc NC is the most flexible and promising model for medical research among various types of protein NCs [20, 21]. To trigger exceptional renal specificity at high accumulation rates, we generated the K3-HBc NCs by inserting K3 peptide (flanked by two glycine-rich flexible linkers) into the surface-exposed spike of HBc NC, and used *E. coli* as a natural bio-factory to produce bioengineered K3-HBc proteins. SDS-PAGE analysis showed the bands corresponding to the indicated molecular weight of HBc-183 and K3-HBc monomer, respectively (Additional file 1: Figure S3A). Western blot analysis using anti-HBc as primary antibody was conducted to validate intrinsic HBc immunogenicity of K3-HBc protein. As shown in Additional file 1: Figure S3B, anti-HBc antibody could recognize HBc-183 but not K3-HBc, indicating the inserted K3 peptide replaced the epitope ofthe MIR region. Meanwhile, CLT nanodots with a diameters of 13.2 ± 2.1 (Fig. 1A, B) had been successfully prepared using a brilliant droplet‐confined/cryodesiccation‐driven crystallization approach [22]. The zeta potential of CLT nanodots was − 21.7 mV, indicating the negatively charged surface of CLT nanodots (Additional file 1: Figure S4). The retained arginine-rich domain of HBc protein provided a positively charged microenvironment inside the nanoparticle of K3-HBc NCs, which facilitated encapsulation of negatively charged CLT nanodots through electrostatic interactions. As a result, CLT nanodots could be efficiently loaded into the cavities through the controlled disassembly and reassembly of K3-HBc NCs. Negatively stained transmission electron microscope (TEM) confirmed monodispersed and well-defined spherical particles of K3-HBc NCs (Fig. 1A, B). Thus incorporation of K3 peptide to the spike of HBc seemed to neither suppress K3-HBc expression nor interfere their self-assembly. The TEM images of the assemble mixture containing K3-HBc subunits and CLT nanodots were shown in Fig. 1A, B. This result demonstrated that ultra-small CLT nanodots were successfully encapsulated into the K3-HBc NCs. The payload was determined to be 1027 ± 39 CLT molecules per one nanocage using UV–vis spectrophotometer. Dynamic Light Scattering (DLS) analysis conducted on K3-HBc NCs and K3-HBc/CLT NCs yielded hydrodynamic diameters of 35.3 ± 1.6 nm and 35.6 ± 2.1 nm, respectively (Fig. 1C). The in vitro drug release profiles of free CLT and K3-HBc/CLT NCs were performed in phosphate buffer at pH 7.4. In Fig. 1D, the release rate of K3-HBc/CLT exhibited a typical sustaining pattern, much slower than that of free CLT, suggesting that K3-HBc/CLT NCs remained stable at physiological pH to avoid premature release.

### In vitro intracellular uptake of K3-HBc/CLT NCs

**Fig. 2:**
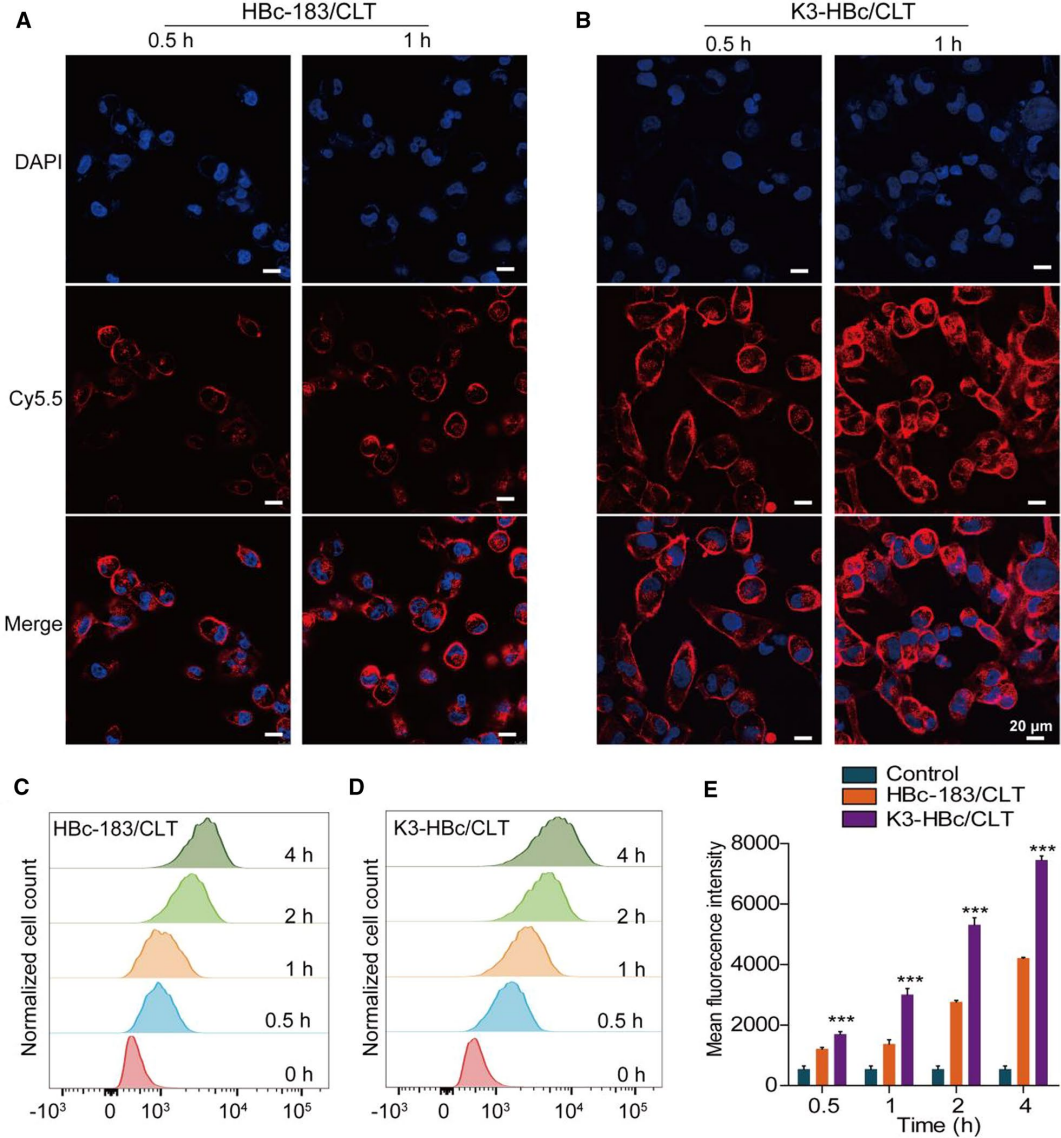
Confocal laser scanning microscope images of HK-2 cells incubated with (**A**) HBc-183/CLT and (**B**) K3-HBc/CLT at different time points (0.5 and 1 h). Representative fluorescence images of nuclei counterstained with DAPI (blue) and HBc-183/CLT and K3-HBc/CLT labeled with Cy5.5 (red), Scale bars, 20 μm. **C**–**E** Flow cytometric analyses of HK-2 cells treated with FITC-labeled HBc-183/CLT or K3-HBc/CLT NCs for 0.5, 1, 2 and 4 h at 37 °C (n = 3) (***p < 0.001)

**Fig. 3:**
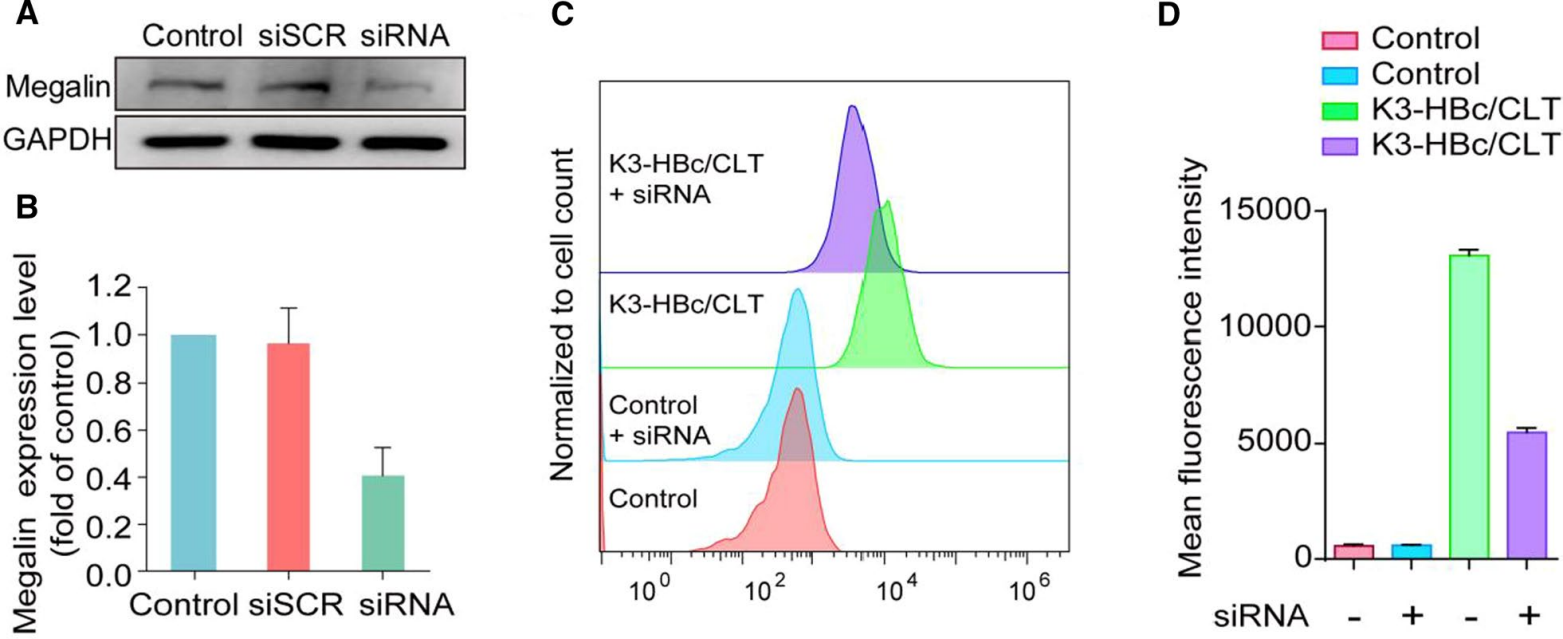
**A** Western blot analysis of siRNA-mediated knockdown of megalin protein in HK-2 cells and (**B**) quantitative analysis of western blot was shown as normalized fold expressions relative to control group using GAPDH as internal control (n = 3). **C**, **D** Flow cytometric analysis of the megalin-knockdown HK-2 cells treated with FITC-labeled K3-HBc/CLT for 1 h at 37 °C (n = 3)

To evaluate the cellular internalization process, Cy5.5-labeled K3-HBc/CLT and HBc-183/CLT NCs were incubated with human proximal tubular epithelial cells (HK-2) cells. As shown by confocal laser scanning microscope images, K3-HBc/CLT NCs were more effectively uptake by HK-2 cells as compared to HBc-183/CLT NCs at 0.5 and 1 h (Fig. 2A, B). The fluorescence signals were also detected using flow cytometry to accurately quantify HBc-183/CLT or K3-HBc/CLT NCs uptake at 0.5, 1, 2 and 4 h (Fig. 2C, D). In Fig. 2E, the median fluorescence intensity values of K3-HBc/CLT treated cells were significantly higher than that of HBc-183/CLT-treated cells at the indicated time points and exhibited a time dependent increase of fluorescence intensity. These findings suggested that K3-HBc/CLT NCs were more efficiently uptake by HK-2 cells. Considering that endocytic receptor megalin is abundantly expressed in renal tubular epithelial cells [23], it is reasonable for K3-HBc to present the high-efficiency endocytosis. Since previous report showed that K3 peptide binds megalin receptor with high affinity [17], it is necessary to confirm whether K3-HBc NCs can effectively bind to HK-2 cells via the specific recognition of megalin receptors. We tested the specificity of K3-HBc/CLT NCs for megalin using siRNA-mediated knockdown (Gene ID: LRP2) in HK-2 cells. Megalin protein status was evaluated 72 h post-siRNA treatment by western blot. The megalin expression level was generally reduced to 59.3 ± 11.9% of control levels estimated (Fig. 3A, B). Remarkably, knockdown of megalin expression in HK-2 cells resulted in 58.4 ± 2.1% suppression of K3-HBc/CLT uptake (Fig. 3C, D).These findings demonstrated that K3-HBc/CLT NCs could be internalized efficiently through the specific interaction between K3 peptide and megalin receptor.

### Biodistribution of K3-HBc/CLT NCs

**Fig. 4:**
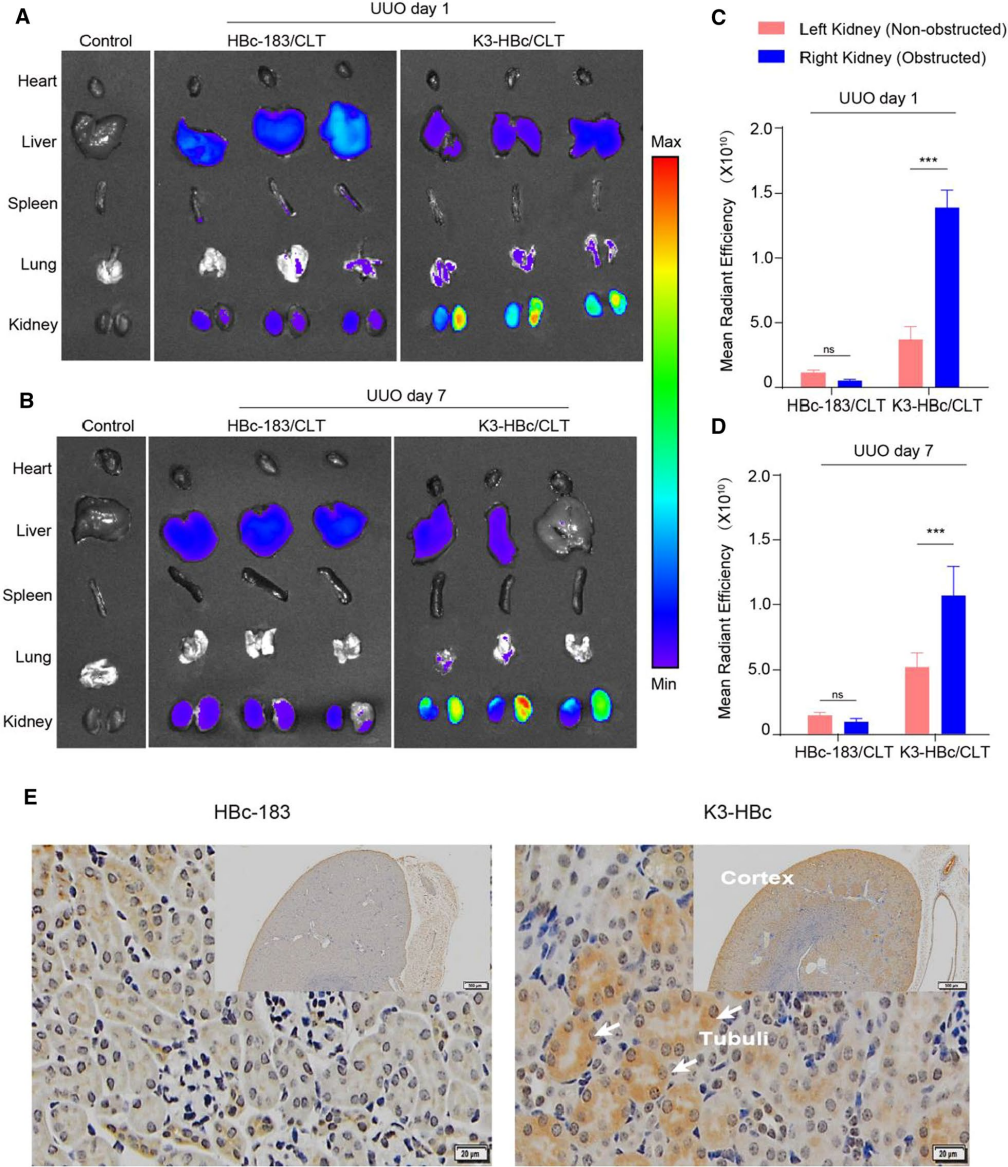
K3-HBc**/**CLT is specifically targeting renal tubular epithelial cells of UUO mice. **A**, **B** Ex vivo fluorescence images of major organs harvested from UUO mice at 1 h post injection of Cy5.5-labeled K3-HBc/CLT or HBc-183/CLT NCs on the first and 7th day. **C**, **D** Fluorescence intensity from excised kidneys (n = 3). **E** Immunohistochemical staining of kidney sections at 4 h after injected with FITC-labeled HBc-183/CLT or K3-HBc/CLT NCs. Scaler bar, 500 μm (upper right panel), 20 μm (left panel) (***p < 0.001)

Next, we investigated the biodistribution of K3-HBc/CLT NCs in vivo. The mice on day 1 or 7 after the UUO were injected with the Cy5.5-labeled K3-HBc/CLT and HBc-183/CLT NCs, respectively. Four hours post injection, the major organs were harvested, and subsequently observed via a near-infrared fluorescent imaging system. The ex vivo biodistribution studies showed significantly different organ distribution patterns for K3-HBc/CLT and HBc-183/CLT NCs (Fig. 4A, B). HBc-183/CLT NCs exhibited prominent uptake in the liver. Meanwhile, the fibrotic kidney uptake was significantly higher in K3-HBc/CLT group than that in HBc-183/CLT group on day 1 or 7 after the UUO (Fig. 4C, D). Obviously, UUO resulted in the destruction of the glomerular filtration barrier, and the presence of leakage and abnormal fenestrae allowed K3-HBc/CLT to cross the barrier and effectively target the tubular epithelial cells. Furthermore, K3-HBc/CLT or HBc-183/CLT NCs were conjugated with fluorescein isothiocyanate (FITC) to identify the targeted cells. Immunohistochemistry examination of kidney showed that FITC-labeled K3-HBc NCs was exclusively found in the renal cortex (Fig. 4E). Higher magnification revealed a specific uptake at the apical side of tubule cells, where the expression of megalin was abundant [24]. This result further revealed that K3-HBc NCs could effectively recognize tubule cells without a loss in specificity.

### Improved efficacy of K3-HBc/CLT NCs in UUO-induced renal fibrosis

**Fig. 5:**
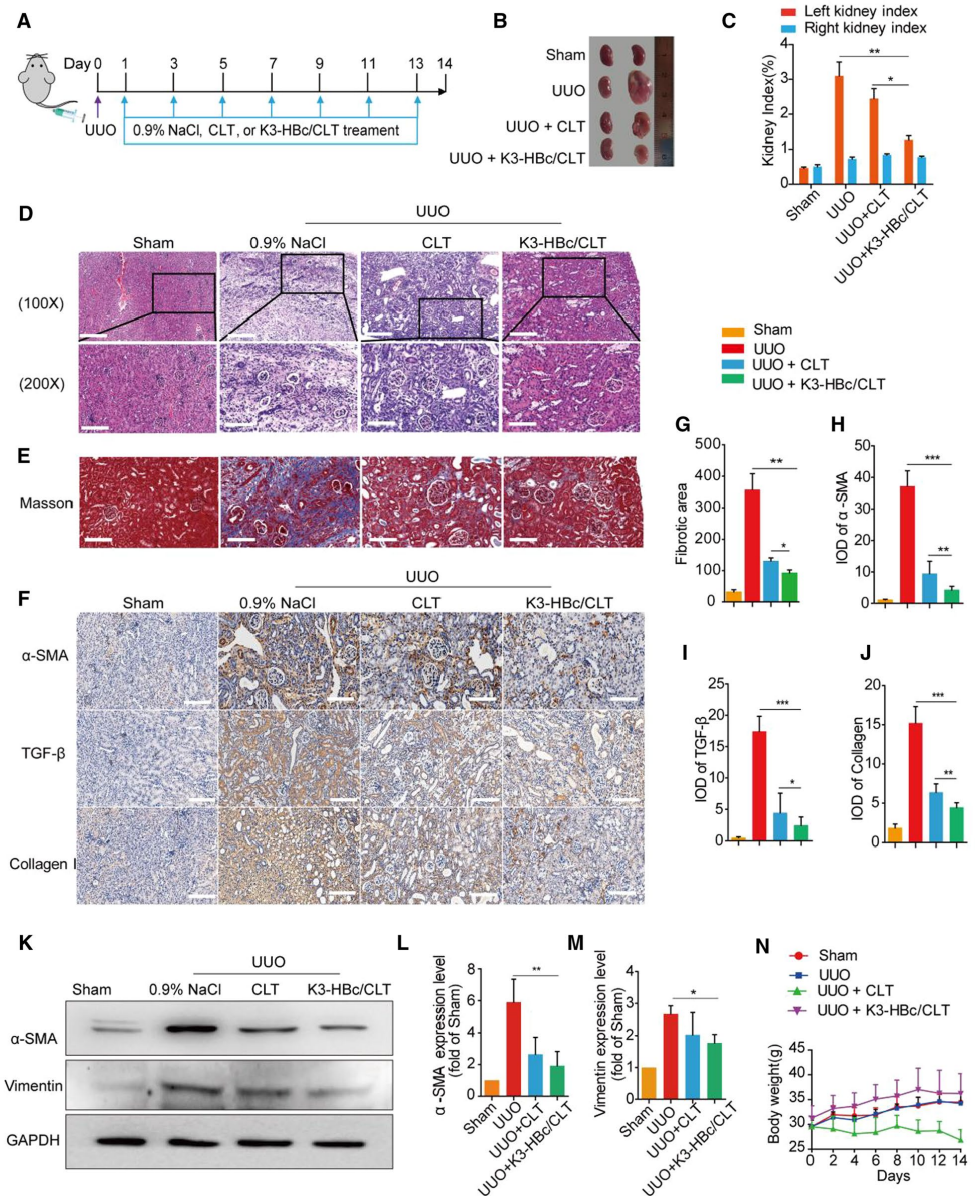
Anti-renal fibrosis effects of K3-HBc/CLT in UUO-induced renal fibrosis. **A** Flow diagram of the treatment of 0.9% NaCl, CLT or K3-HBc/CLT against the UUO mice model. **B** The gross-morphological images of kidney tissue from each group. **C** Kidney index of the UUO mice were measured (obstructed kidney weight /body weight × 100 or non-obstructed kidney/body weight × 100) (n = 5). **D** Representative images of H&E staining of kidney sections after different treatments as indicated. **E** Representative images of Masson’s trichrome staining of kidney sections after different treatments as indicated. **G** Quantification of Masson’s trichrome positive area of collagen-like matrix deposition (n = 6). **F** Representative micrographs of the expression and distribution of α-SMA, TGF-β and collagen I in kidney tissues sections using immunohistochemical staining and the semi-quantitative IOD analyses of (**H**) α-SMA, (**I**) TGF-β and (**J**) collagen I (n = 6). **K** Western blot analyses of α-SMA and Vimentin in mice on day 14 after treatment with CLT or K3-HBc/CLT and quantitative analyses of protein levels of (**L**) α-SMA and (**M**) Vimentin (n = 3). **N** The body weights within 2 weeks measured every two days in different groups (n = 5). Scale bar, 100 μm (*p < 0.05, **p < 0.01, ***p < 0.001)

To examine the therapeutic efficacy of K3-HBc/CLT NCs, we investigated their ability to reduce extensive accumulation of extra-cellular matrix within the interstitium in UUO-induced renal fibrosis. We first confirmed that K3-HBc NCs could not prevent UUO-induced renal fibrosis (Additional file 1: Figure S5). The induction of renal fibrosis and treatment in mice were schematically presented in Fig. 5A. The renal protective effect of K3-HBc/CLT NCs was observed by the morphology of kidney in each group (Fig. 5B). The major characteristics observed in obstructed kidneys of mice were fluid retention and swollen. Moreover, the left kidney index (1.26 ± 0.12%) in UUO + K3-HBc/CLT group was remarkably lower than that in UUO + 0.9% NaCl group (3.10 ± 0.39%) (*p* < 0.01) or in UUO + CLT group (*p* < 0.5) (Fig. 5C). Obviously, K3-HBc/CLT appeared to prevent obstructed kidney enlargement more effectively than free CLT.

To evaluate the impact of K3-HBc/CLT NCs on TGF-β-induced EMT processes in vitro, we measured the expression levels of E-cadherin, fibronectin and vimentin in TGF-β-induced HK-2 cells (Additional file 1: Figure S6). As expected, the E-cadherin expression was decreased, while the fibronectin and vimentin expression level were increased relative to those in control cells. However, cells treated with TGF-β + CLT or TGF-β + K3-HBc/CLT NCs exhibited restoration of those pro-fibrotic markers, indicating that CLT and K3-HBc/CLT NCs could prevent the TGF-β-induced EMT processes.

The pathological and molecular changes of kidney in different groups were further evaluated. Histological investigation revealed that marked tubular dilation and atrophy, interstitial matrix deposition caused by UUO were greatly ameliorated by K3-HBc/CLT NCs (Fig. 5D). Remarkably, interstitial collagen deposition in obstructed kidney was significantly reduced by K3-HBc/CLT NCs (Fig. 5E). Likewise, the fibrotic area of kidney (92.90 ± 9.02) in UUO + K3-HBc/CLT group was further less than that (131.40 ± 9.19) in UUO + CLT group (Fig. 5G). The immunostaining of fibrotic markers in kidney revealed that K3-HBc/CLT-treated mice exhibited a more significant reduction in obstruction-induced α-SMA, TGF-β expressions or collagen Ι accumulation, compared with CLT-treated mice (Fig. 5F, H–J). Similar results were observed in the western blot studies (Fig. 5K). The expressions of α-SMA and vimentin in obstructed kidney were all significantly downregulated in UUO + K3-HBc/CLT group (Fig. 5L, M). Taken together, these results demonstrated that K3-HBc/CLT NCs exhibited a better anti-fibrotic effect than CLT on renal fibrosis.

We investigated the systemic toxicity of CLT or K3-HBc/CLT NCs in mice on day 14 after treatment. We noticed that mice underwent a steady body weight loss during treatment with free CLT (Fig. 5N). On the contrary, the K3-HBc/CLT-treated mice gained weight similar to the mice in sham group. K3-HBc/CLT NCs displayed an advantage over free CLT concerning the body weight loss. H&E staining revealed that K3-HBc/CLT NCs effectively prevented serious heart, liver, and brain damage caused by free CLT. No obvious toxic effects were observed in the spleen and lung in UUO + K3-HBc/CLT group (Additional file 1: Figure S7). Moreover, K3-HBc/CLT NCs significantly decreased the elevated blood levels of aminotransferase (AST), aminotransferase (ALT), lactate dehydrogenase (LDH-L), and creatinine (CREA) caused by UUO, but had no significant differences in blood urea nitrogen (BUN) and total bilirubin (TBiL) levels (Additional file 1: Figure S8). In detail, K3-HBc/CLT NCs could decrease the ALT level from 77.25 ± 1.01 U/L in UUO + 0.9% NaCl group to 52.84 ± 1.40 U/L (*p* < 0.001) as well as the AST level from 112.82 ± 8.86U/L in UUO + 0.9% NaCl group to 69.66 ± 3.78 U/L (*p* < 0.01). The elevation of CREA in UUO + 0.9% NaCl group (24.91 ± 0.67 mmol/L) could be decreased by K3-HBc/CLT (18.45 ± 1.65 mmol/L) (*p* < 0.05). The serum level of LDH-L was 335.56 ± 21.68 U/L in UUO + K3-HBc/CLT group, which was significantly lower than that in UUO + 0.9% NaCl group (458.57 ± 8.77 U/L) (*p* < 0.01). We also evaluated the systematic toxicity of K3-HBc NCs in vivo. As shown in Additional file 1: Figure S9, K3-HBc-treated mice maintained the level of ALT and AST within the normal range. Besides, the H&E staining of sliced organs showed no sign of early lesions in K3-HBc group. The profile of cytokines such as Tumour necrosis factor α (TNF-α), Interleukin-12 (IL-12), or Interleukin-6 (IL-6) in the serum of healthy mice treated with K3-HBc NCs was analyzed. There was no significant difference between the treated and the control groups (Additional file 1: Figure S10). These results indicated that K3-HBc NCs could serve as a safe carrier. Taken together, K3-HBc/CLT NCs could improve the CLT therapeutic effect without severe toxic effect.

### K3-HBc/CLT NCs exerts marked anti-fibrotic effect through inhibiting cell senescence in UUO mice 

**Fig. 6:**
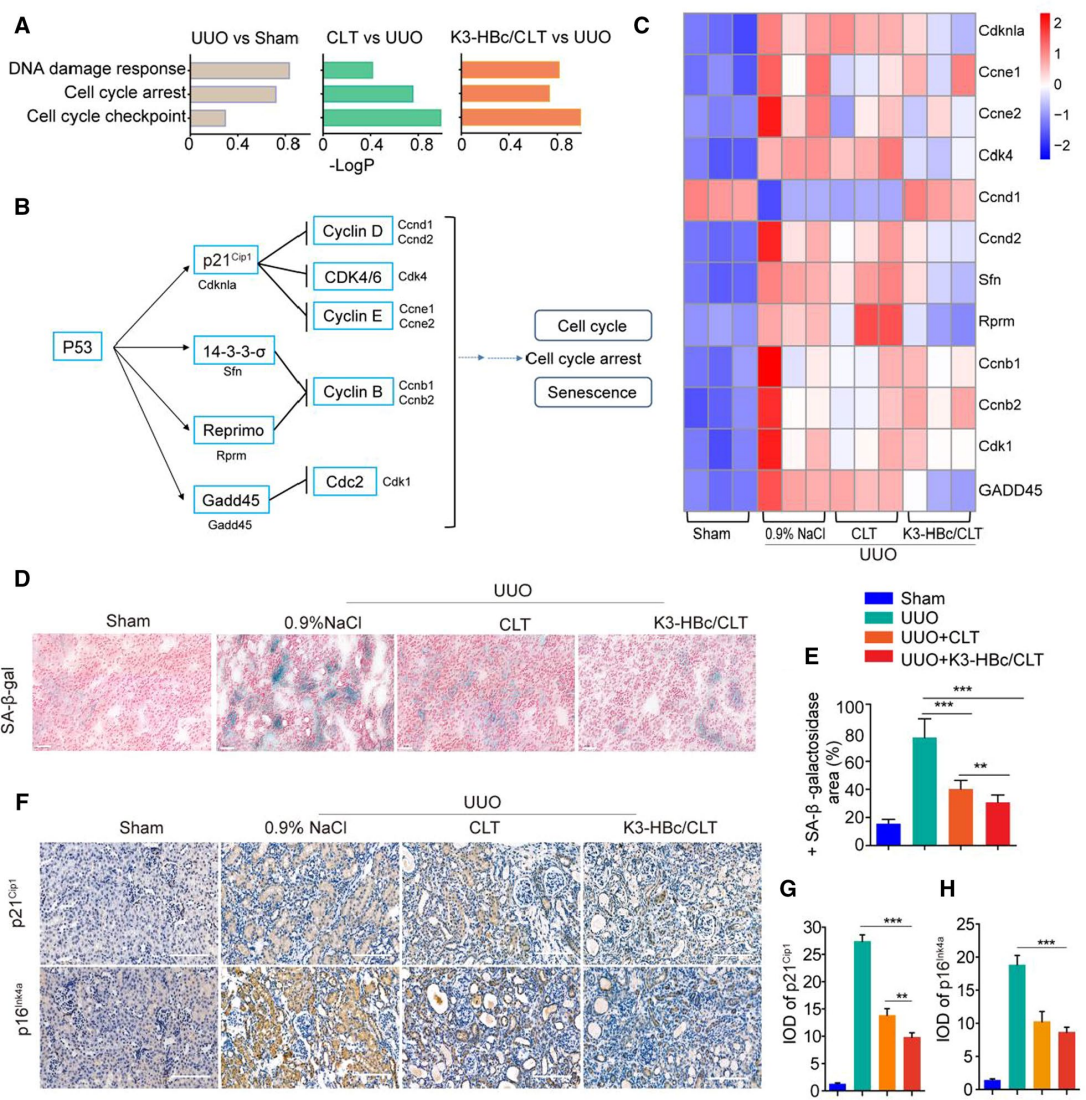
K3-HBc/CLT alleviated tubular cell senescence in vitro and in vivo. **A** Predominant biological process analyses of differentially expressed genes using GO enrichment. **B** The p53 signaling pathways roadmap and the related genes of cell cycle and cell senescence. **C** Heatmaps of the most differentially expressed genes in Sham, UUO, CLT and K3-HBc/CLT groups. **D** Representative renal SA-β-gal staining in sham, UUO, UUO + CLT and UUO + K3-HBc/CLT mice. Scale bar, 50 μm. **E** Percentage of SA-β-gal positive area in four groups. **F** Representative micrographs of the expression and distribution of p21^Cip1^ and p16^Ink4a^ in the kidney sections using immunohistochemical staining and the semi-quantitative IOD analyses of (**G**) p21^Cip1^ and (**H**) p16^Ink4a^ after different treatments as indicated (n = 3). Scale bar, 100 μm (**p < 0.01, ***p < 0.001)

Previous report indicated that the cell senescence of tubular epithelial cells contributed to renal fibrosis [25, 26]. Meanwhile, stable cell cycle arrest, a defining feature of senescence [27], is a functional consequence of EMT program during fibrotic injury [28]. To better understand the anti-fibrotic role of cell scenecence in response to K3-HBc/CLT NCs, high throughput RNA-seq was performed in each group. We compared biological processes corresponding with cell senescence including DNA damage response, cell cycle arrest and cell cycle checkpoint in all three comparisons by gene ontology (GO) enrichment analysis (Fig. 6A). In addition, persistent DNA damage response (DDR) due to chronic genomic stress or telomere attrition leads to activation of p53, which in turn activates p21^Cip1^ to initiate cell cycle arrest [29, 30]. The core signaling pathways were related with p53, cell cycle and senescence (Fig. 6B). By Kyoto Encyclopedia of Genes and Genomes (KEGG) enrichment analysis, the heatmap of regulated genes expression related to p53, cell cycle and senescence in each group was shown in Fig. 6C. 11 individual genes in these categories, including Cdknla, Ccne1, Ccne2, Cdk4, Ccnd1, Ccnd2, Sfn, Rprm, Ccnb1, Ccnb2, Cdk1 and GADD45, were significantly regulated in UUO + 0.9% NaCl group compared to sham group. The changes in expression levels for GADD45, Sfn, and Cdk4 genes revealed by quantitative real-time PCR (qPCR) were similar to those determined by the RNA-Seq analysis (Additional file 1: Figure S11). K3-HBc/CLT NCs largely reversed the alterations in expression levels of the genes caused by UUO. Therefore, it was reasonable to speculate that these cell senescence and cell cycle genes acted vigorously in K3-HBc/CLT NCs mediated anti-fibrotic effect.

To solidify this conclusion, we examined the effect of CLT and K3-HBc/CLT NCs on cell senescence. Increased β-galactosidase activity and elevated transcript or protein levels of p16^Ink4a^ and p21^Cip1^ are key molecular characteristics of senescent cells [31]. In vitro senescent HK-2 cells induced by Ang-II were incubated with CLT or K3-HBc/CLT NCs, following β-galactosidase staining [32]. Cell senescence in UUO kidkey as indicated by SA-β-Gal staining was decreased by CLT and K3-HBc/CLT NCs (Fig. 6D, E). Next, we examined the expression of cell senescence marker by immunocytochemistry. The expression of p21^Cip1^ and p16^Ink4a^ were remarkably elevated in UUO + 0.9% NaCl group, but K3-HBc/CLT NCs and CLT could significantly downregulate these expressions in obstructed kidney (Fig. 6F). Semi-quantitative analyses showed that K3-HBc/CLT NCs could downregulate expression of p16^Ink4a^ and p21^Cip1^ in obstructed kidney nobly, and K3-HBc/CLT-treated mice expressed p21^Cip1^ protein at lower level compared with CLT-treated mice (Fig. 6G, H). Based on these results, we concluded that K3-HBc/CLT NCs exerted protective effect against renal fibrosis through ameliorating cell senescence.

## Discussion

Regardless of the underlying initiating cause, renal fibrosis leads to loss of function and end-stage renal failure [33], requiring all-life dialysis or kidney transplantation. Considering the difficulty of dealing with the underlying process, a specific and effective anti-fibrotic therapy is urgently needed. Here, we found that CLT showed potent protection of kidney function and effects against renal fibrosis, consistant with previous report [34]. Targeted delivery of CLT to tubular epithelial cells using K3-HBc NCs has demonstrated to circumvent restraints such as systemic toxic effects. Li et al. reported that targeted loading of CLT by CREKA-coupled liposomes to interstitial myofibroblast allowed the drug to alleviate UUO-induced renal fibrosis [35]. Meanwhile, targeted delivery of celastrol to mesangial cells is also effective against mesangio proliferative glomerulonephritis [12]. Comparing to these findings, K3-HBc/CLT NCs in our study were expected to see further improvement in the efficacy for renal fibrosis, since tubular epithelial cells could synthesize and secrete varieties of bioactive molecules that drive interstitial inflammation and fibrosis [8, 36, 37].

Precise delivery of therapeutic agent to tubular epithelial cells by K3-HBc NCs to prevent maladaptive repair would be a promising strategy for halting the fibrotic process [38]. The main features of K3-HBc NCs include: (i) superb biocompatibility and biodegradability; (ii) well-defined biological structure; (iii) tailored modifications by synthetic biology strategy; (iv) capability of self-assembly and a large internal cavity; (v) suitability for imparting non-natural functions by chemical functionalization. K3-HBc/CLT NCs made the special target-cell contact by binding to megalin via the K3 peptide [17]. In addition, megalin possesses an intracellular domain with a signaling domain for clathrin-mediated endocytosis and allows for kidney-specific delivery in fibrosis [39].As a result, K3-HBc/CLT NCs binding efficiently triggered tubular epithelial cells uptake through receptor-mediated endocytosis [40]. Our results demonstrated K3-HBc/CLT NCs could precisely mimic the structure of the authentic virus, specifically interacted with tubular epithelial cells in a virus-like manner.

The design of K3-HBc NCs is a valuable strategy to enhance CLT specificity for therapeutic applications. Targeted delivery of CLT by K3-HBc/CLT NCs to tubular epithelial cells significantly alleviated collagen deposition and myofibroblast infiltration in UUO-induced renal fibrosis. Moreover, K3-HBc/CLT NCs improved the pharmacokinetics and bioavailability of CLT with reduced drug accumulation in non-targeted tissue and organs, thereby minimizing CLT-related toxicity [41]. Importantly, future clinical application of CLT seems to be limited by its poor water solubility [42]. With K3-HBc NCs, CLT could been given by intravenous injection, making substantial progress in the future clinical application of CLT. In addition, many kidney-targeted drug delivery systems employ synthetic carriers, such as limposomes and micells, have been extensively investigated [43, 44]. However the high cost, low purity, and variations remain the major obstacles to clinical translation. The well-defined K3-HBc NC can be produced with high yields in *E. coli* expression system without any additional chemical modification, which makes it easy to scale up and may be more conducive to clinical transformation.

Transcriptome sequencing by RNA-seq provided novel perspectives to better understand the molecular mechanisms on attenuation of renal fibrosis by K3-HBc/CLT NCs. In particular, we were able to find strong up-regulation of cell senescence and cell cycle genes in UUO-injured kidney. Previous studies have suggested that the structural and functional alterations caused by cellular senescence in tubule cells may play an essential role in the initiation and/or progression of renal fibrosis [45]. The transcriptome analyses showed that there was a close lineage relationship between cell senescence and UUO-injured kidney. Senescence can produce various pro-fibrotic and pro-inflammatory cytokines to promote renal fibrosis [8]. Pharmaceutical blockade of these effects or elimination of senescent cells can counteract fibrosis [46, 47]. In our study, K3-HBc/CLT NCs as well as CLT effectively prevented the UUO-induced up-regulation of cell senescence signaling. Hence, tubule-specific K3-HBc/CLT NCs extraordinarily prevented an early event that occurred before the onset of renal fibrosis via inhibition of cell senescence.

## Conclusions

In summary, we designed an intriguing new method to deliver CLT specifically to tubular epithelial cells by K3-HBc NCs via binding to the megalin receptor. K3-HBc/CLT NCs significantly alleviated renal fibrosis without severe systemic toxicity. Importantly, p21^Cip1^ and p16^Ink4a^ pathway was found to be the novel anti-fibrotic molecular basis of K3-HBc/CLT NCs to prevent UUO-induced premature senescence in tubular epithelial cells. The targeted delivery of anti-fibrotic drugs by using K3-HBc/CLT NCs provided an effective and promising therapeutic strategy for renal fibrosis.

## Methods

### Materials

Celastrol (98.0%) was purchased from Sichuan Weikeqi Biotech Co., Ltd. FITC and Cy5.5-NHS were purchased from MedChemExpress. Pluronic F-127 was purchased from Sigma-Aldrich. All the chemicals used in this study were of analytical grade.

### Preparation of K3-HBc/CLT NCs

The amino acid sequence of HBc-183 is the same as the previously reported [48]. K3 peptide accompanied with two glycine-rich linkers was incorporated to the surface-exposed major immunodominant loop region of HBc-183 (between the residues 78 and 81) to yield K3-HBc NCs. The objective plasmid carrying the K3-HBc gene was purchased from Shanghai Generay Biotech Co., Ltd. The corresponding vectors were transformed into *E. coli* strain BL21 (DE3). The expression and purification of K3-HBc protein were performed as the previous description [49].

Ultra-small CLT nanodots were fabricated by droplet-confined crystallization during the freeze-drying of frozen emulsion [22]. CLT-containing dichloromethane was mixed with an aqueous solution containing F127 (1.0 wt%), and then converted into an O/W emulsion by sonification. The emulsion was immediately immersed in liquid nitrogen and rapidly frozen, followed by freeze-drying. The resulted CLT nanodots were harvested and washed by centrifugation-redispersion cycles to remove free F127.

The bioengineered K3-HBc NCs were reversibly disassembled with 8 M urea at 25 °C for 3 h. After the dissociation, ultra-small CLT nanodots were added and the solution was gently stirred for 2 h. Then, the reassembly of K3-HBc NCs was conducted by dialysis in an assembling buffer at 4 °C and free CLT nanodots was removed by SuperdexTM-75 column. To calculate the loading capacity, the concentration of CLT was determined by UV–vis spectrophotometer.

The morphology of ultra-small CLT nanodots, K3-HBc NCs and K3-HBc/CLT NCs were observed using a transmission electron microscope (TEM, JEM-1400, JEOL). The hydrodynamic diameter sizes of NCs and zeta potential of CLT nanodots were respectively determined by Zeta Sizer (Nano Series, Malvern). Besides, the diameter distributions of CLT nanodots and NCs were obtained through counting at least 100 particles in TEM images.

The FITC-labeled HBc-183 or K3-HBc NCs were performed as the previous description [50]. Briefly, FITC was dissolved in DMSO, and then were mixed with HBc-183 or K3-HBc NCs and gently vortexed for 2 h in the dark at 25 °C. The mixtures were dialyzed in PBS buffer at 4 °C for 48 h to remove unreacted FITC molecules. Cy5.5-NHS was labeled to HBc-183 or K3-HBc NCs in the same methods with FITC labeling.

### In vitro* drug release*

Dialysis bags (MWCO = 3000 Da) containing 2 mL of K3-HBc/CLT and CLT solution (CLT, 2 mg) were separately submerged in release medium(50 mL) at 37 °C while stirring at 100 r/min. At predetermined time points (0.25, 0.5, 1, 2, 4, 8, 12, 24, 36 and 48 h), 1 mL aliquots of the solution were withdrawn to determinate the released drug. Then an equal volume of fresh buffer was added to keep the volume constant and to ensure sink conditions throughout the whole process. These samples were determined by high performance liquid chromatography (HPLC) to calculate the cumulative amount of released drug and plot the percentage of drug released.

### Animals model and therapeutic experiments

The male Sprague Dawley rats (6–8 weeks) and male BALB/C mice (6–8 weeks) were purchased from animal center in Third Military Medicine University (Chongqing, China). All animal care and experiments were conducted in compliance with the requirements of the National Act on the use of experimental animals (China) and were approved by the Institutional Animal Care and Ethic Committee of Third Military Medicine University.

UUO was a well-established experimental model of renal fibrosis [51]. Animals were anesthetized with 1% pentobarbital (10 μL g^−1^), the left ureter was exposed by a tilted incision which near the lower edge of the left rib 1 cm, and then it was obstructed by two-point ligations with 4–0 silk sutures. The abdominal incision was sealed with the same silk suture, and animals were returned to the cages. Sham-operated mouse underwent the same procedure except ligation.

The rats were randomized into three groups: the sham group treated with PBS, the UUO group treated with 0.9% NaCl, and UUO + CLT group received free CLT (1 mg kg^−1^). PBS, 0.9% NaCl or free CLT was injected intraperitoneally every two days. The body weights were recorded every two days. All animals were sacrificed on day 14. Kidney index from each group were measured (obstructed kidney weight/body weight × 100% or non-obstructed kidney/body weight × 100%). The heart, liver, spleen, lung kidney and brain were harvested immediately. Tissues were washed, fixed and stained with H&E. Histological lesions were observed under the microscope (Olympus BX51, Japan).

To study the therapeutic effect of K3-HBc/CLT in the UUO model, 24 mice were randomly divided into four groups (n = 6): sham, UUO + 0.9% NaCl, UUO + CLT and UUO + K3-HBc/CLT groups. The mice were treated with CLT (1 mg kg^−1^) by intraperitoneal injection or K3-HBc/CLT (CLT, 1 mg kg^−1^) by tail vein injection. Both CLT and K3-HBc/CLT were administered every two days. The mice were treated with an equal volume of 0.9% NaCl in UUO + 0.9% NaCl group. All mice were sacrificed on day 14. The organs were harvested for H&E staining. Before animals were sacrificed, blood was collected and centrifuged at 2500 rpm for 15 min. The serum was measured on a chambray 240 automatic biochemical analyzer (Radyto, China) for the following analytes: BUN,CREA, AST, ALT, TBIL and LDH-L, which indicates renal, liver and heart functions, respectively.

### Cell culture and TGF-β-induced EMT processes in vitro

HK-2cells were purchased fromStem Cell Bank, Chinese Academy of Sciences. The HK-2 cells were cultured in Dulbecco’s modified Eagle’s medium (DMEM)/F12 medium (Hyclone, SH30023.01, Thermo Scientific, USA) supplemented with 10% fetal bovine serum (ScienCell, Carlsbad, CA, USA), 100 U mL^−1^ penicillin/streptomycin (Beyotime, Shanghai, China). Cells were cultured in a humidified atmosphere containing 5% CO_2_ at 37 °C.

Serum-starved HK-2 cells were pre-incubated with or without the CLT (500 nM) or K3-HBc/CLT (CLT, 500 nM) for 1 h for the in vitro experiments. Then the cells were stimulated with TGF-β1 (5 ng mL^−1^) at the indicated time points. HK-2 cell morphology was observed by phasecontrast microscopy and the cell lysate was harvested for further study.

### Cellular uptake of K3-HBc NCs in vitro

HK-2 cells were seeded onto sterilized microscope coverslips placed in 6-well plates at a density of 2 × 10^5^ cells/well and incubated in DMEM medium supplemented with 10% fetal bovine serum for 24 h at 37 °C with 5% CO_2_. Afterwards, the cells were treated with Cy5.5-labeled K3-HBc/CLT NCs or HBc-183/CLT NCs under the equivalent NCs concentration (20 μg mL^−1^) and co-incubated for 0.5 and 1 h. Subsequently, the cells were washed 5 min with PBS for three times, fixed with cold 4% paraformaldehyde for 20 min and washed three times with PBS. DAPI was used to stain the nuclei of the cells. Finally, the cells were observed with a confocal laser scanning microscope (Zeiss LSM 700, Zeiss, Thornwood, Germany).

The cellular uptake of FITC-labeled-K3-HBc/CLT NCs or HBc-183/CLT NCs in HK-2 cells was quantified by flow cytometry. Briefly, HK-2 cells (1 × 10^5^ cells/well) were seeded in 12-well plates and incubated for 24 h. Then, cells were incubated with FITC-labeled K3-HBc/CLT NCs or HBc-183/CLT NCs under the equivalent concentration (20 μg mL^−1^). At predetermined times (0.5, 1, 2 and 4 h), cells were washed with PBS and detached using trypsin. Finally, cells were suspended in cold PBS, which was then immediately analyzed by BD FACS Verse flow cytometer (BD Biosciences, San Jose, CA, USA).

The siRNA transfection experiments were performed as reported previously [52]. The reagents for silencing of gene expression were obtained from Sangon Biotechnology. We employed siRNAs specifically targeting mRNA for humanmegalin and a nontargeting scramble-sequence siRNA (siSCR) as a negative control. HK-2 cells were transfected with siSCR or siRNA targeting LRP2 for 72 h. Then cells were incubated with FITC-labeled K3-HBc/CLT NCs for 1 h, the cellular uptake efficiency was analyzed by flow cytometry.

### Distribution of K3-HBc/CLT NCs in vivo

Mice on day 1 or day 7 after the UUO received Cy5.5-labeled HBc-183/CLT or K3-HBc/CLT NCs via the tail vein. Four hours post injection, the mice were sacrificed and the major organs including heart, liver, spleen, lung and kidney were excised and imaged using an imaging system (IVIS Spectrum, Caliper LifeSciences, USA).

### SA-β-galactosidase staining

The ability of K3-HBc/CLT to inhibited cell senescence in UUO mice kidney was measured by SA-β-Gal staining. Frozen mouse kidney sections (4 μm thickness) were used for detection of SA-β-Gal. The activity of SA-β-Gal was analyzed with SA-β-Gal staining kit (Beyotime, Shanghai, China) according to the manufacturer’s instructions.

### Western blot analysis

Total proteins were isolated from kidney and HK-2 cells and quantified as described previously [53]. Protein samples (30 μg/lane) were resolved using sodium dodecyl sulfate–polyacrylamide electrophoresis and then transferred onto polyvinylidene difluoride membranes (PVDF, Millipore, USA). The membranes were incubated at 4 °C overnight with α-SMA (1:300, Abcam, Cambridge, MA, USA), fibronectin (1:300, Santa Cruz, CA, USA), vimentin (1:500, Santa Cruz, CA, USA), E-cadherin (1:1000, Proteintech, Wuhan, China), Megalin (1:100, Santa Cruz, CA, USA) and HBc (1:1000, Abcam, Cambridge, MA, USA), followed by incubation with horseradish peroxidase-conjugated goat anti-mouse (1:10,000, Santa Cruz, CA, USA) or goat anti-rabbit IgG secondary antibodies (1:10,000, Santa Cruz, CA, USA) at room temperature for 1 h. The immunoblots were imaged by the chemiluminescence western blot detection system (Bio-Rad ChemiDoc MP, California, USA) with GAPDH (1:5000, ZENBIO, Chengdu, China) as the loading control.

### qPCR analysis

Total RNA was extracted from the obstructed kidney mice treated with 0.9%NaCl, CLT, or K3-HBc/CLT for 14 days using Trizol reagent (TaKaRa, Japan) according to the manufacturer’s recommendations. Subsequently, 1 μg RNA was used to reverse transcribed cDNA by PrimeScript RT reagent Kit with gDNA Eraser (TaKaRa, Japan). cDNA abundance was de-termined by qPCR with SYBRPRIME qPCR Kit (Bioground, China). The following primers were used: Cdknla forward, 5′-tgatctgctgctcttttcc-3′, and reverse, 5′-tacattcccttccagtcca-3′; Gadd45 forward, 5′-agaagaccgaaaggatgga-3′, and reverse, 5′-cgtaatggtgcgctgac-3′; Sfn forward, 5′-cgacagtgctggggaag-3′, and reverse, 5′-ccaaggtgtggctgaaca-3′; Rprm forward, 5′-acgcaaacctgtcggagt-3′, and reverse, 5′-tgccacctgctgctgtat-3′; Cdk4 forward, 5′-gctgaaattggtgtcggt-3′, and reverse, 5′-cctccagaatccttaaca-3′; GAPDH forward, 5′-ggttgtctcctgccgacttca-3′, and reverse, 5′-tggtccagggtttcttactcc-3′. GAPDH serves as an endogenous control.

### Histology and immunohistochemical staining

Kidney tissues harvested from animals on day 14 were fixed in 4% paraformaldehyde, embedded in paraffin and cut into 4 μm thick per section. The sections were stained with H&E and Masson’s trichrome staining. The collagen deposition in the obstructed kidney tissue was assessed by Masson’s trichrome staining. At least 10 randomly selected cortical fields were observed under the microscope (Olympus BX51, Japan) and the renal fibrotic area was semi-quantitated using image-pro plus 6.0 software.

The immunohistochemistry experiment was performed as previously described [54]. Briefly, sections mounted on slides were blocked with 5% BSA for 1 h and incubated over night at 4 °C with primary antibodies against α-SMA (1:200), collagen I (1:500, Santa Cruz, CA, USA), TGF-β (1:200), p16 (1:500) and p21 (1:500). The slides were then stained with a goat anti-rabbit IgG secondary antibody (1:50, Beyotime, jiangsu, China). The results were analyzed using a 3,3′-diaminobenzidine (DAB) assay kit (Servicebio, Wuhan, China). The slides were visualized by a microscope (Olympus BX51, Japan) and were semi-quantitated based on the intensity and spread of positive staining in terms of IOD using image-pro plus 6.0 software.

To examine the localization of K3-HBc NCs in the kidney, immunohistochemical studies were performed in BALB/c mice. The FITC-labeled K3-HBc NCs or HBc-183 NCs was administered to mice via tail vein injection. At 1 h post injection, mice were sacrificed and harvested kidneys were fixed in 4% paraformaldehyde for 24 h. The primary antibody was rabbit anti-FITC antibody (1:500, Sangon Biotech, Shanghai, China). The immunohistochemical staining and analysis was performed as described above.

### RNA-seq and bioinformatic analysis

RNA-seq experiment was performed as previous descriped [55]. Briefly, RNA was isolated from kidney tissues in sham, UUO, UUO + CLT and UUO + K3-HBc/CLT groups using the Trizol kit according to the manufacturer ‘s instructions, respectively. Sample integrity, quality and purity were determined accordingly. cDNA synthesis was performed from DNase1 treated RNA samples using ImProm-II Reverse Transcriptase (Promega Corp., Madison, WI). Sequencing libraries were generated using NEBNext^®^ UltraTM RNA Library Prep Kit for Illumina^®^ (NEB, USA) following manufacturer’s recommendations. The sequencing service is performed by Novogene Corporation (Beijing, China). Raw reads were generated using an Illumina Novaseq 6000 platform by paired-end sequencing. After quality filtering, the clean reads were mapped onto the mouse reference genome and the read count for each gene was derived from the mapping results obtained by Feature Counts. All read counts were normalized to fragments per kilo bases per million mapped reads (FPKM). DEseq2 was used to determine differential expressions. Transcripts with an adjusted p value, 0.05 were accepted as being differential. Differentially expressed genes from the different comparisons and the subjected biological process and molecular functional pathways were analyzed using the Gene Ontology, Kyoto Encyclopedia of Genes and Genomes (KEGG) and Reactome biochemical pathway databases.

### Statistical analysis

Quantitative results were expressed as mean ± SEM. Statistical differences among groups were checked by One-way ANOVA followed by Newman-Keuls multiple comparisons test or Student’s unpaired two-tailed t test from GraphPad Prism 5.0 (GraphPad Software, San Diego, CA, USA). *p* < 0.05 was considered statistically significant.

## Supplementary Information

**Additional file 1: Figure S1. **CLT suppressed UUO-induced renal fibrosis. **Figure S2**. Representative H&E-stained images of heart, liver, spleen, lung and brain on day 14 after treatment. **Figure S3**. (A) SDS-PAGE analysis of K3-HBc or HBc-183. (B) Western blot analysis of HBc-183 or K3-HBc with antibody against HBc. **Figure S4**. Zeta potential of ultra-small CLT nanodots. **Figure S5**. K3-HBc NCs were administered to the mice in UUO + K3-HBc NCs group by tail vein injection at a dosage of 9.49 mg/kg every other day starting immediately after UUO operation. **Figure S6**. Anti-EMT effects of CLT orK3-HBc/CLT in vitro. Serum-starved HK-2 cells were incubated with CLT or K3-HBc/CLT (500 nM) for 1 h and then stimulated with TGF-β1 (5 ngmL-1) for 48h. **Figure S7**. Representative H&E stained images of the organs harvested from the mice after various treatments. **Figure S8**. Blood biochemistry analyses of the mice after treatment with CLT or K3-HBc/CLTfor 14 days. **Figure S9**. (A) Blood biochemistry analyses of the healthymice after treatment with K3-HBc for 14 days. The results showed mean and standard deviation of AST, ALT, BUN, CREA, LDH-L, TBiL (n = 3). (B) Representative H&E stained images of the organs harvested from the mice after treatment with K3-HBc for 14 days. Scale bar=100 μm. **Figure S10**. Serum cytokine analysis in mice. **Figure S11**. mRNA levels of (A) Cdknla, (B) GADD45, (C) Rprm, (D) Sfn, and (E) Cdk4 were measured by qPCR in obstructed kidney from the mice treated with 0.9%NaCl, CLT, or K3-HBc/CLT for 14 days (n = 3).

## Data Availability

All data related to the manuscript are available in the manuscript in the form of figures.

## Abbreviations

ALT: Aminotransferase
α-SMA: α-Smooth muscle actin
AST: Aminotransferase
BUN: Blood urea nitrogen
CKD: Chronic kidney disease
CLT: Celastrol
CREA: Creatinine
EMT: Epithelial–mesenchymal transition
FITC: Fluorescein isothiocyanate
HBc: Hepatitis B core protein
HK-2: Human proximal tubular epithelial cells
H&E: Hematoxylin and eosin
IOD: Integrated optical density
LDH-L: Lactate dehydrogenase
NCs: Nanocages
TBIL: Total bilirubin
TEM: Transmission electron microscope
TGF-β: Transforming growth factor-beta
TWHF: Tripterygiumwilfordii Hook F
UUO: Unilateral ureteral obstruction
SA-β-gal: Senescence-associated β-galactosidase
TNF-α: Tumour necrosis factor α
IL-12: Interleukin-12
IL-6: Interleukin-6
qPCR: Quantitative real-time PCR
