# Supplementary material for: Tubule-specific protein nanocages potentiate targeted renal fibrosis therapy
Source: J Nanobiotechnology. 2021 May 26;19:156. doi: 10.1186/s12951-021-00900-w (PMC8157627; doi:10.1186/s12951-021-00900-w)
Supplement: Supplementary file 1 — Additional file 1: Figure S1. CLT suppressed UUO-induced renal fibrosis. Figure S2. Representative H&E-stained images of heart, liver, spleen, lung and brain on day 14 after treatment. Figure S3. (A) SDS-PAGE analysis of K3-HBc or HBc-183. (B) Western blot analysis of HBc-183 or K3-HBc with antibody against HBc. Figure S4. Zeta potential of ultra-small CLT nanodots. Figure S5. K3-HBc NCs were administered to the mice in UUO + K3-HBc NCs group by tail vein injection at a dosage of 9.49 mg/kg every other day starting immediately after UUO operation. Figure S6. Anti-EMT effects of CLT orK3-HBc/CLT in vitro. Serum-starved HK-2 cells were incubated with CLT or K3-HBc/CLT (500 nM) for 1 h and then stimulated with TGF-β1 (5 ngmL-1) for 48h. Figure S7. Representative H&E stained images of the organs harvested from the mice after various treatments. Figure S8. Blood biochemistry analyses of the mice after treatment with CLT or K3-HBc/CLTfor 14 days. Figure S9. (A) Blood biochemistry analyses of the healthymice after treatment with K3-HBc for 14 days. The results showed mean and standard deviation of AST, ALT, BUN, CREA, LDH-L, TBiL (n = 3). (B) Representative H&E stained images of the organs harvested from the mice after treatment with K3-HBc for 14 days. Scale bar=100 μm. Figure S10. Serum cytokine analysis in mice. Figure S11. mRNA levels of (A) Cdknla, (B) GADD45, (C) Rprm, (D) Sfn, and (E) Cdk4 were measured by qPCR in obstructed kidney from the mice treated with 0.9%NaCl, CLT, or K3-HBc/CLT for 14 days (n = 3). [file 12951_2021_900_MOESM1_ESM.docx]

**Additional file 1**

Tubule-specific Protein Nanocages Potentiate Targeted Renal Fibrosis Therapy

Xuan Zhang^1#^, Qian Chen^2#^, Liyuan Zhang^1^, Haiping Zheng^3^, Chunjie Lin^4^, Qunfang Yang^1^, Tao Liu^1^, Haigang Zhang^1^, Xiaohong Chen^1^, Lei Ren^5^*, and Wenjun Shan^1,6,^*

1. Department of Pharmacology, College of Pharmacy and Laboratory Medicine, Army Medical University (Third Military Medical University), Chongqing, 400038, P. R. China

2. Biomedical Analysis Center, College of Basic Medicine, Army Medical University (Third Military Medical University), Chongqing, 400038, P. R. China

3. School of Medicine, Xiamen University, Xiamen, 361102, P. R. China

4. School of Life Sciences, Xiamen University, Xiamen, 361102, P. R. China

5. Department of Biomaterials, College of Materials, Xiamen University, Xiamen 361005, P. R. China

6. School of Medicine, Huaqiao University, Quanzhou, 362021, P. R. China

^#^These authors contributed equally to this work.

**Corresponding author:* Lei Ren, [renlei@xmu.edu.cn](mailto:renlei@xmu.edu.cn);

**Corresponding author:*Wenjun Shan, [wjshan@tmmu.edu.cn](mailto:wjshan@tmmu.edu.cn)

Keywords: protein nanocage, celastrol, target therapy, cell senescence, renal fibrosis


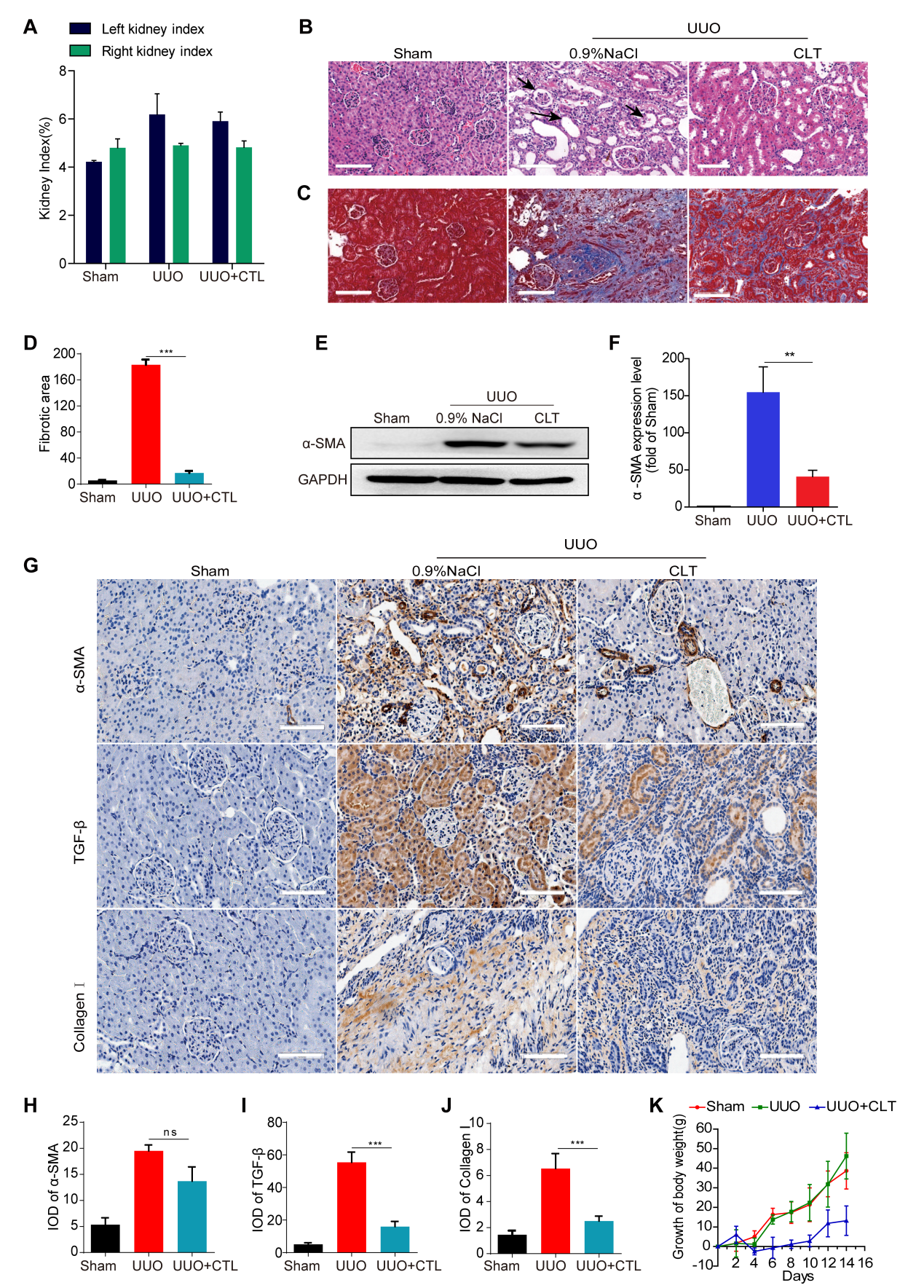


**Figure S1.** CLT suppressed UUO-induced renal fibrosis. **(**A) Kidney index from each group were measured. (B) Representative images of H&E staining of kidney sections. Black arrows indicated the glomerular atrophy and the tubular dilation. Scale bar, 100 μm. (C) Representative images of Masson’s trichrome staining of kidney sections. The blue areas represented the collagen I deposition. Scale bar, 100 μm. (D) Planimetric quantifications of fibrotic area of kidneys in different groups. (E) Western blot analysis of renal α-SMA protein, and (F) quantitative analysis was shown as normalized fold expressions relative to sham group using GAPDH as internal control (n = 5). (G) Representative kidney sections of immunohistochemical staining for α-SMA, TGF-β and collagen Ⅰ in different groups. Scale bar, 100 μm. The semi-quantitative IOD analyses of (H) α-SMA, (I) TGF-β and (J) collagen Ⅰ in the kidney sections (n = 5). (K) The growth of body weight was measured every two days in different groups (n = 5).(***p*< 0.01, ****p*< 0.001).­


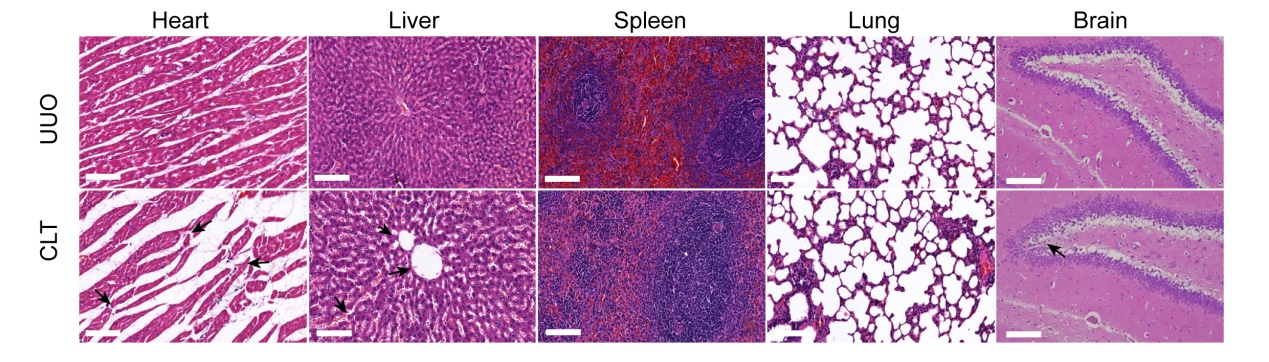


Figure S2. Representative H&E-stained images of heart, liver, spleen, lung and brain on day 14 after treatment. The cardiomyocyte atrophy (black arrows), the dilation of blood sinuses (black arrows) and the pyknosis of neurons (black arrows) were observed, respectively. Scale bar, 100 μm.


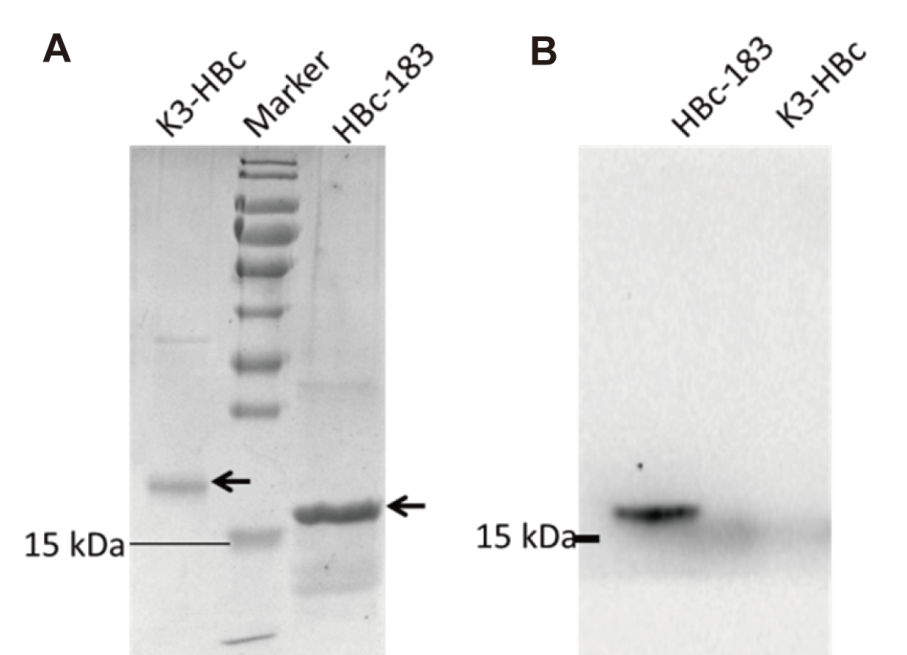


**Figure S3.** (A) SDS–PAGE analysis of K3-HBc or HBc-183. (B) Western blot analysis of HBc-183 or K3-HBc with antibody against HBc.

**
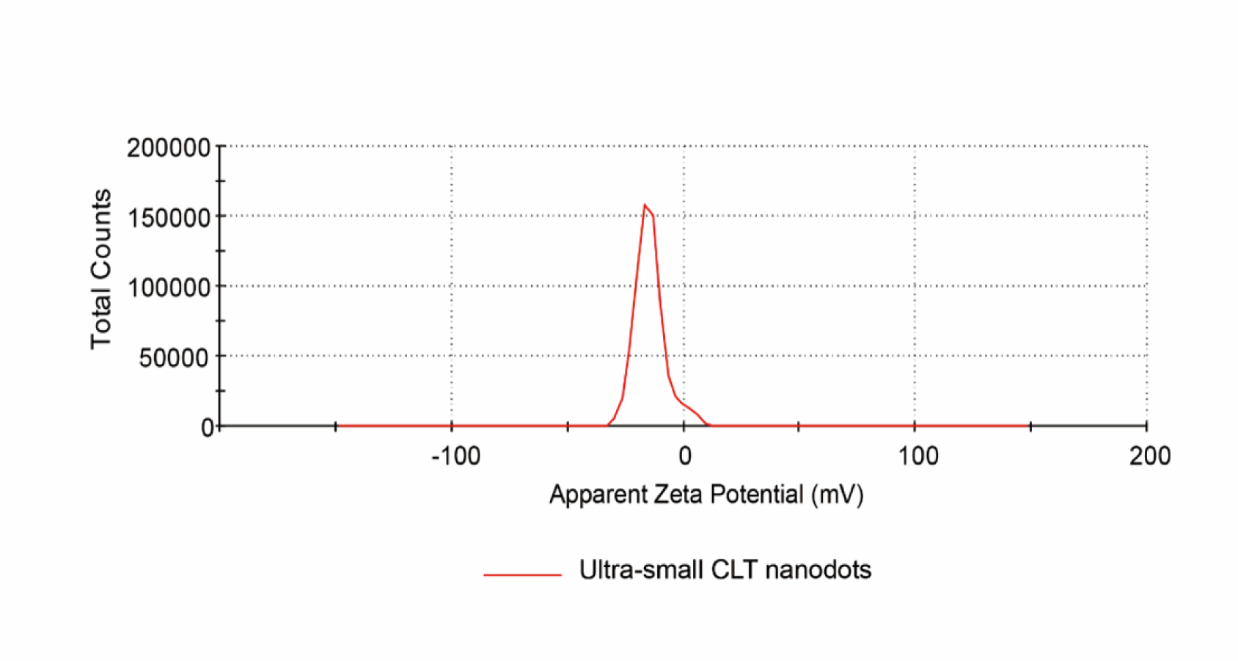
**

**Figure S4.**Zeta potential of ultra-small CLT nanodots.


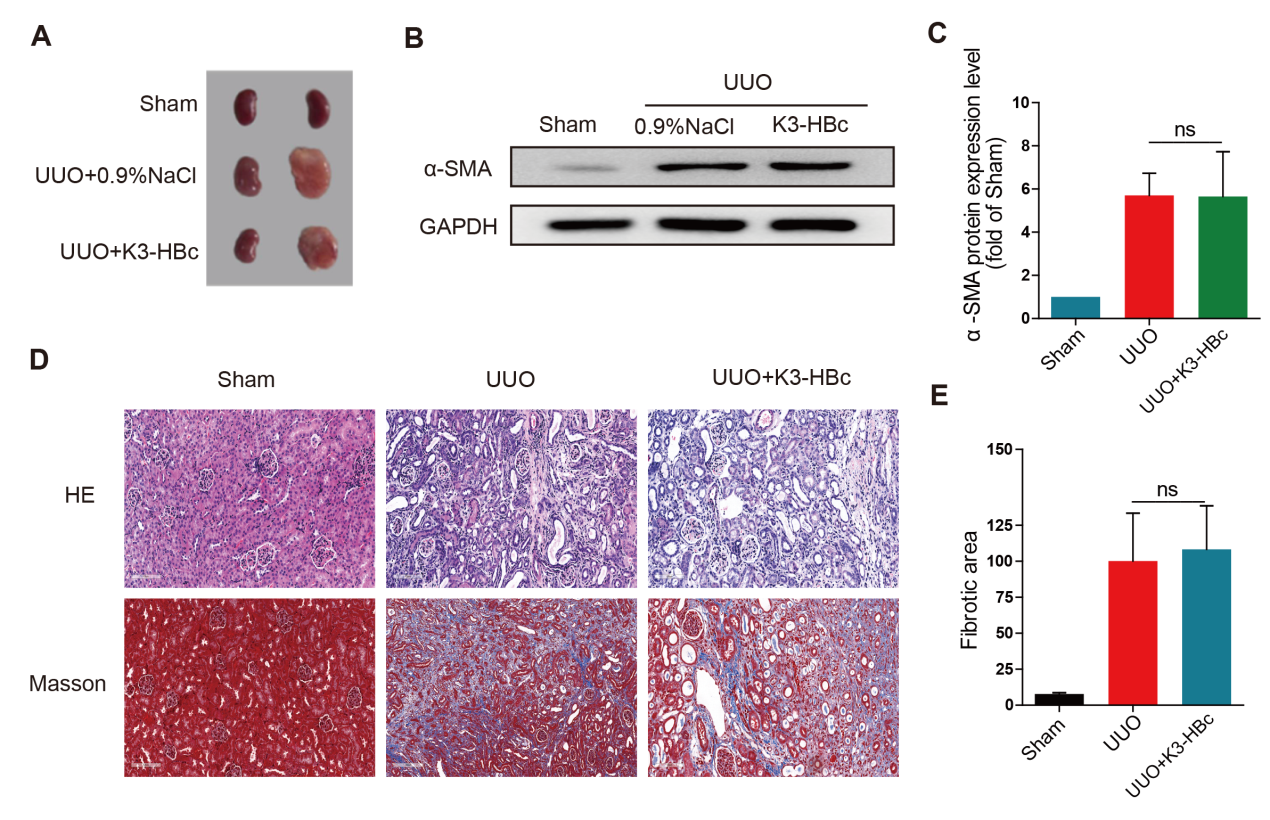


**Figure S5.** K3-HBc NCs were administered to the mice in UUO + K3-HBc NCs group by tail vein injection at a dosage of 9.49 mg/kg every other day starting immediately after UUO operation. (A) The gross-morphological images of kidney from each group. (B) Western blot analysis of α-SMA in mice on day 14 after treatment with K3-HBc and quantitative analysis of protein level of (C) α-SMA (n = 3). (D) Representative images of H&E staining and Masson’s trichrome staining of kidney sections after different treatments as indicated. Scale bar, 100 μm. (E) Quantification of Masson’s trichrome positive area of collagen-like matrix deposition (n = 3). The obstructed kidney of mice in UUO group or UUO + K3-HBc NCs group was fluid retention and swollen comparing with normal kidney on day 14. The EMT-induced α-SMA and collagen deposition in UUO + K3-HBc NCs group were not significantly different from that in UUO group, which suggested that K3-HBc NCs could not prevent UUO-induced renal ﬁbrosis.


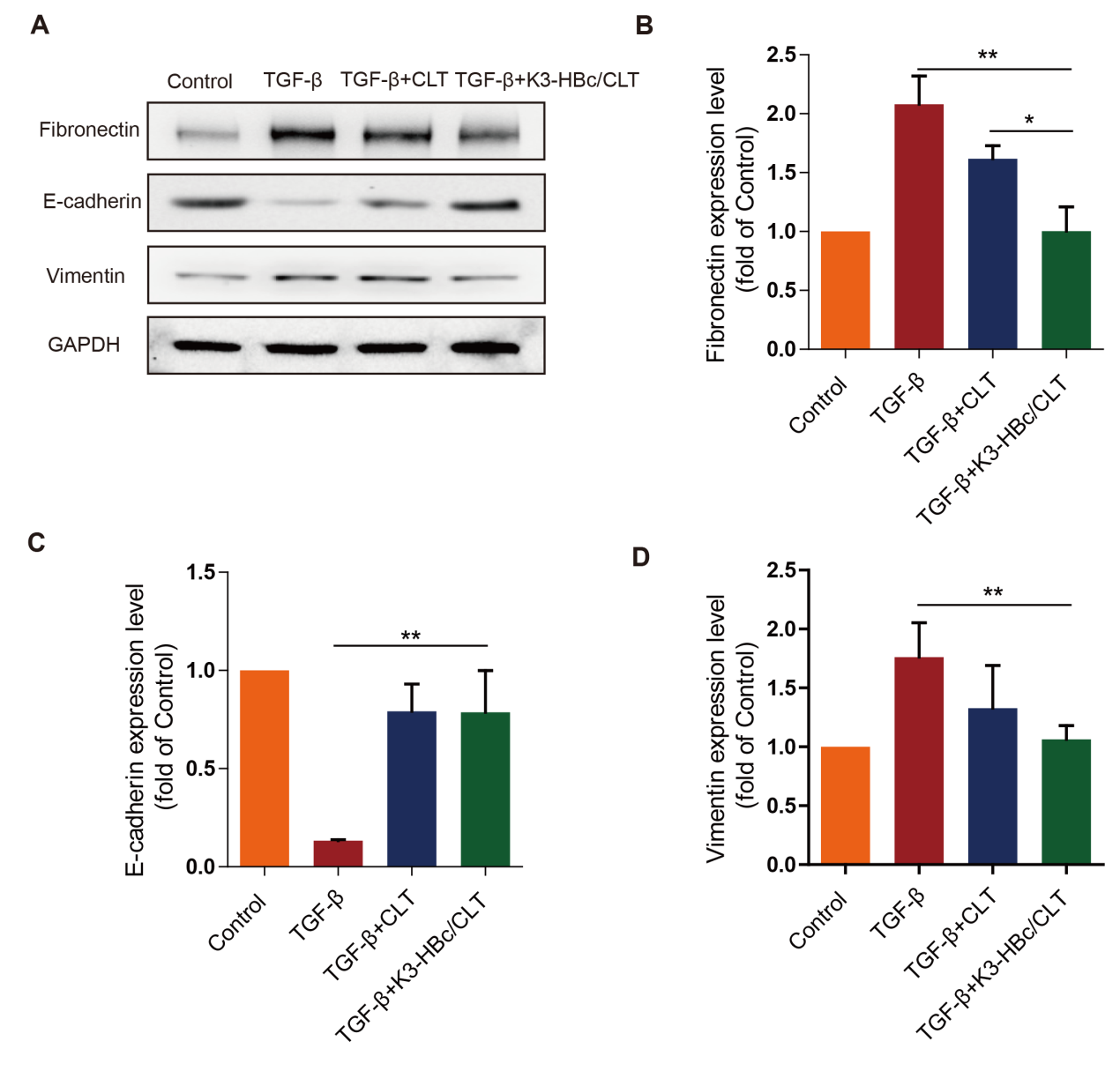


**Figure S6**. Anti-EMT effects of CLT orK3-HBc/CLT *in vitro*. Serum-starved HK-2 cells were incubated with CLT or K3-HBc/CLT (500 nM) for 1 h and then stimulated with TGF-β1 (5 ngmL^-1^) for 48h. (A) Representative western blot analyses of fibronectin, E-cadherin and vimentin expression in HK-2 cells. (B-D) Quantification of fibronectin, E-cadherin and vimentin expression levels in HK-2 cells. (n = 3). (**p*< 0.05, ***p*< 0.01).


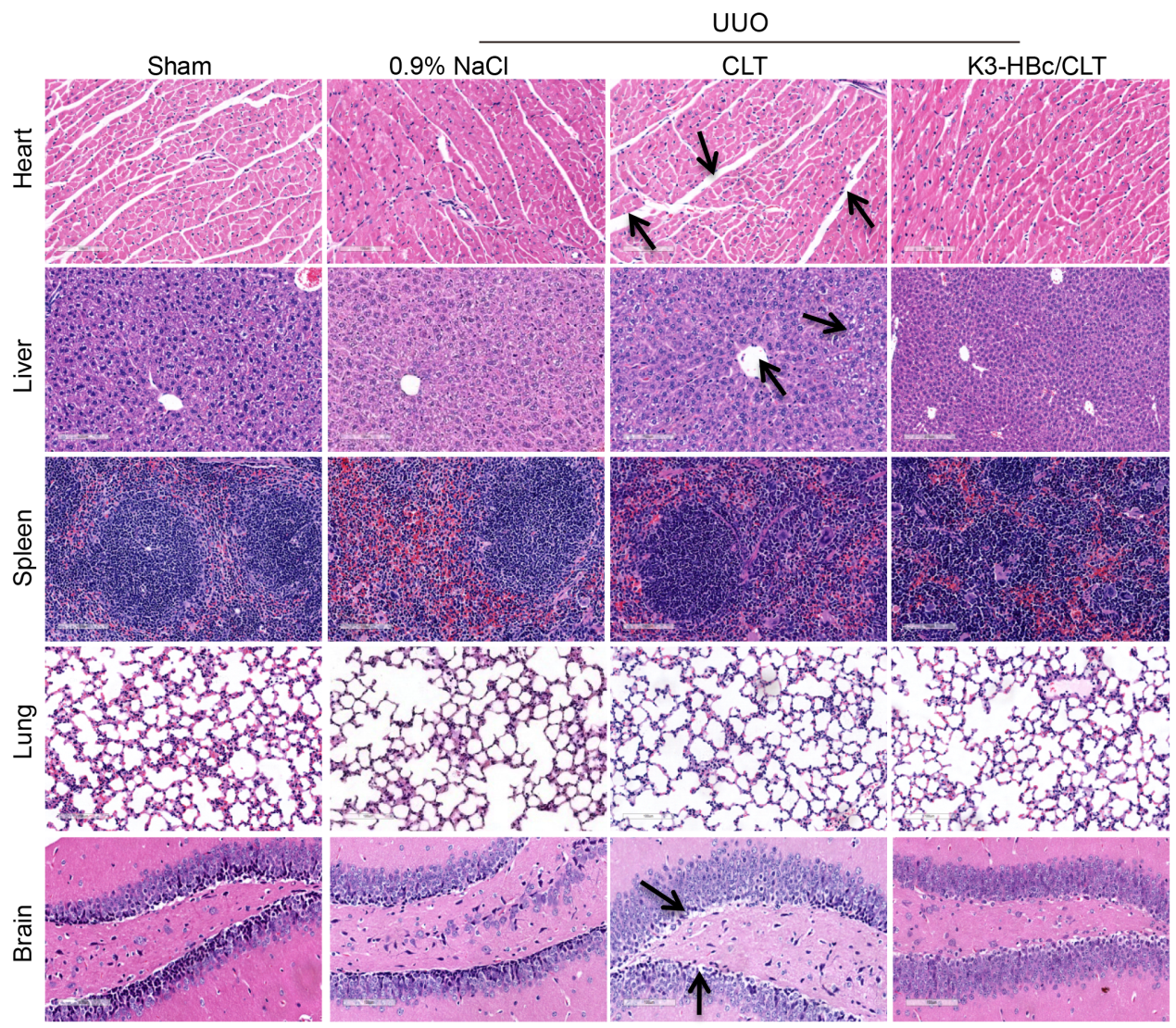


**Figure S7**. Representative H&E stained images of the organs harvested from the mice after various treatments. The cardiomyocyte atrophy (black arrows), the dilation of blood sinuses (black arrows) and the pyknosis of neurons (black arrows) were observed, respectively. Scale bar=100 μm.


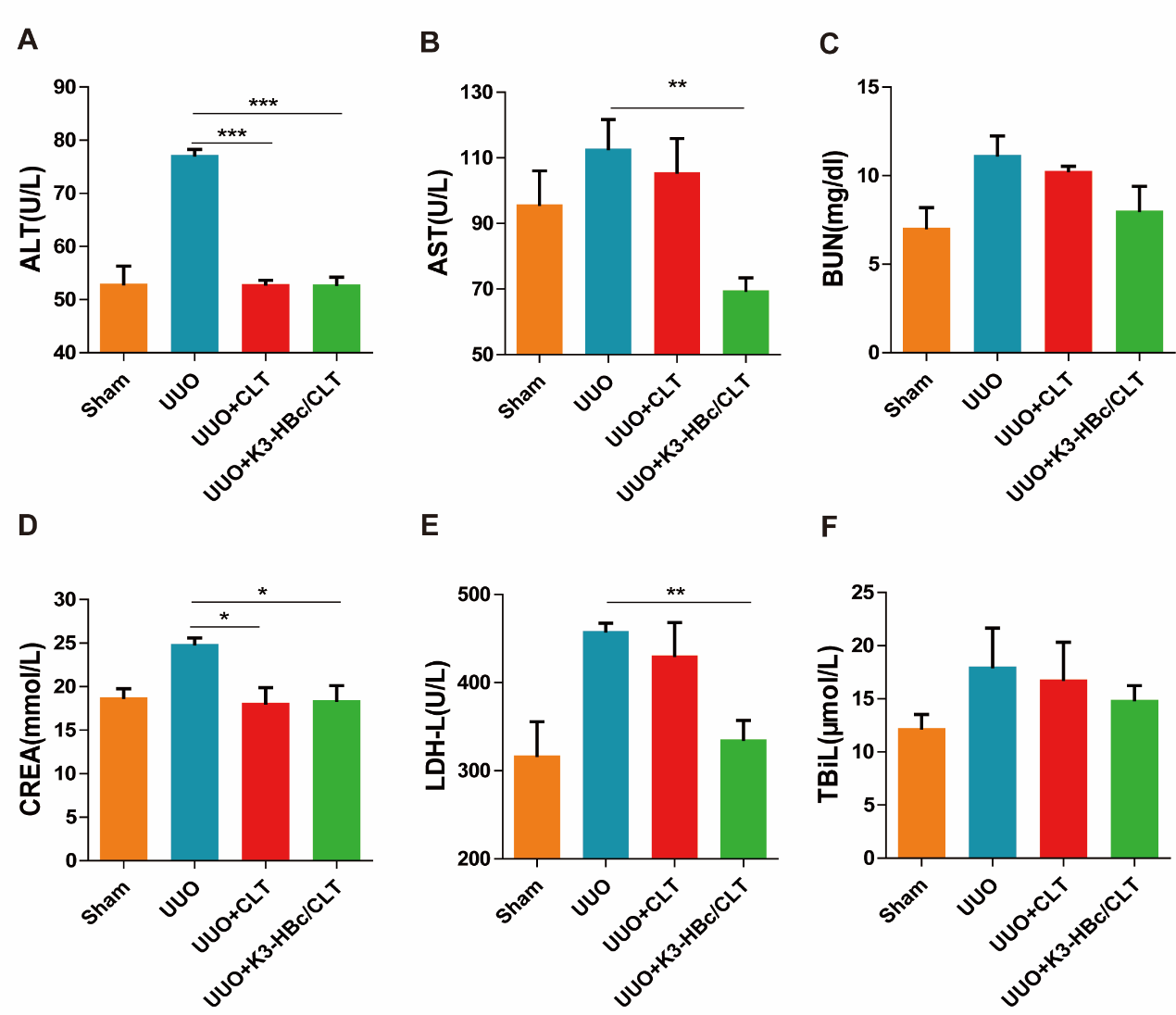


**Figure S8**. Blood biochemistry analyses of the mice after treatment with CLT or K3-HBc/CLTfor 14 days. The results showed mean and standard deviation of AST (A), ALT (B), BUN (C), CREA (D), LDH-L (E), TBiL (F). (n = 5). (**p*< 0.05, ***p*< 0.01 and ****p*<0.001).


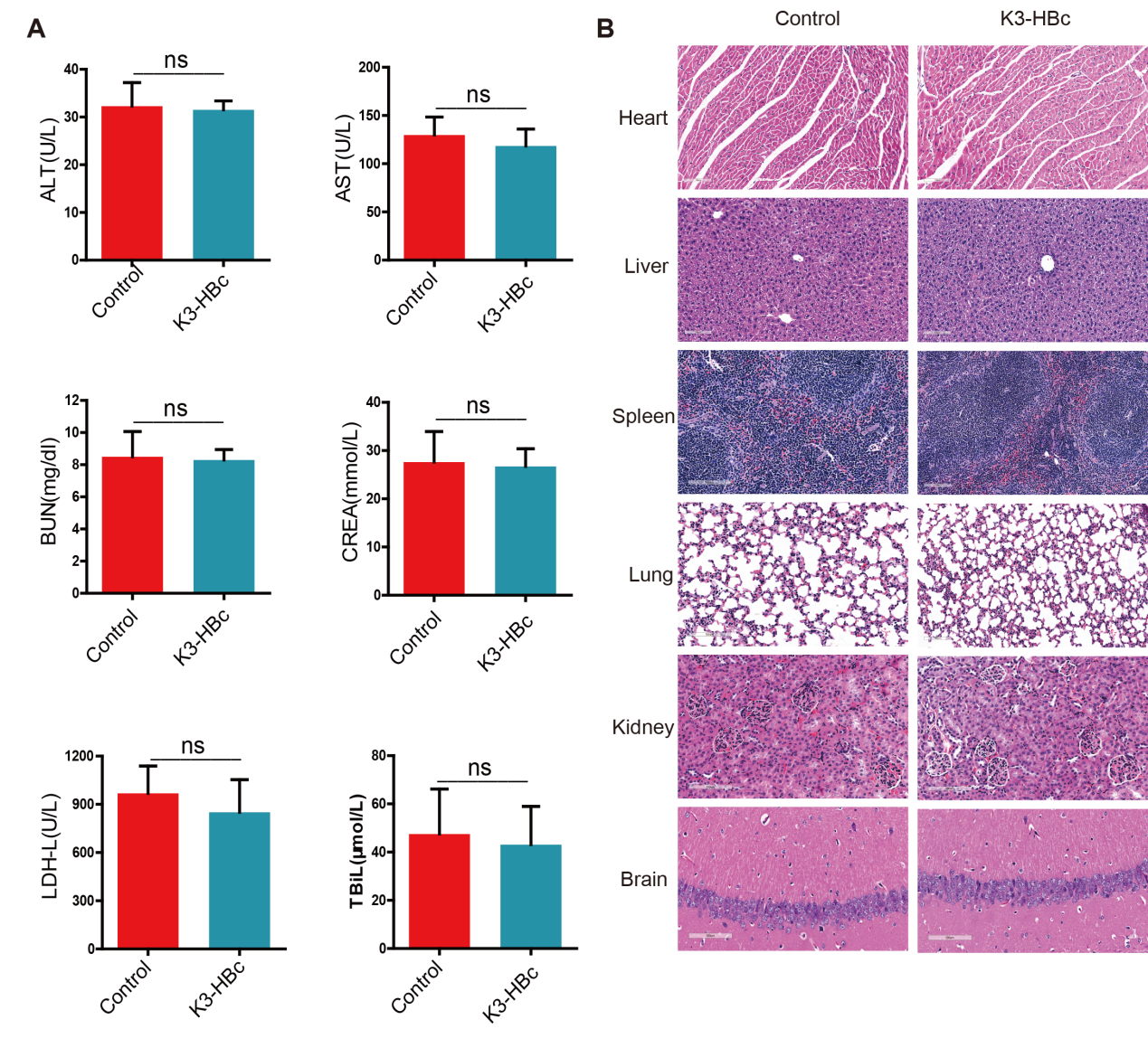


**Figure S9.** (A) Blood biochemistry analyses of the healthymice after treatment with K3-HBc for 14 days. The results showed mean and standard deviation of AST, ALT, BUN, CREA, LDH-L, TBiL (n = 3). (B) Representative H&E stained images of the organs harvested from the mice after treatment with K3-HBc for 14 days. Scale bar=100 μm.


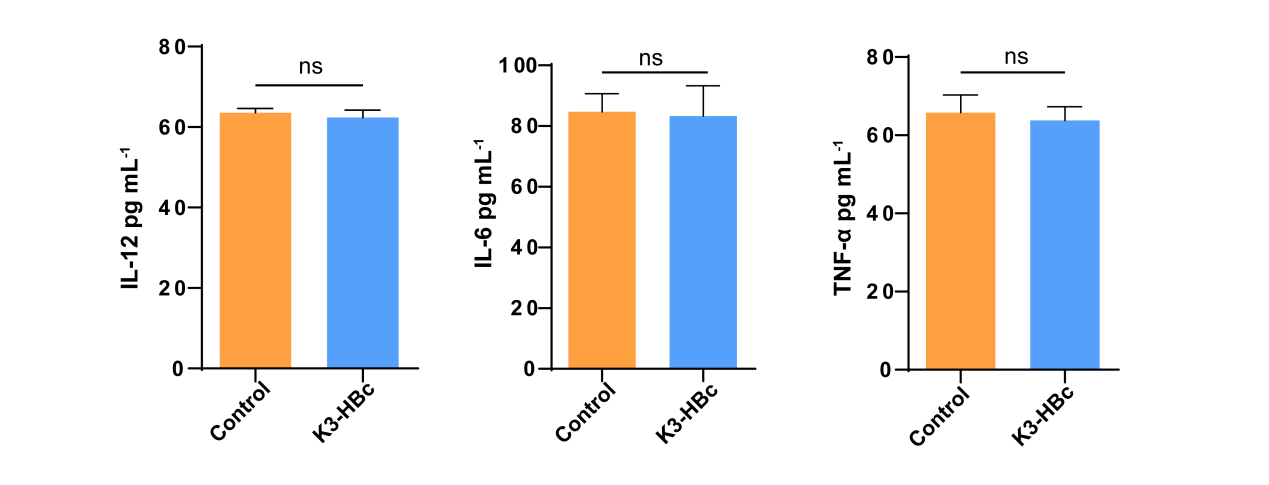


**Figure S10.** Serum cytokine analysis in mice. Sera were obtained from the mice after treatment with K3-HBc for 14 days, and cytokine concentrations were measured by ELISA (n = 5).


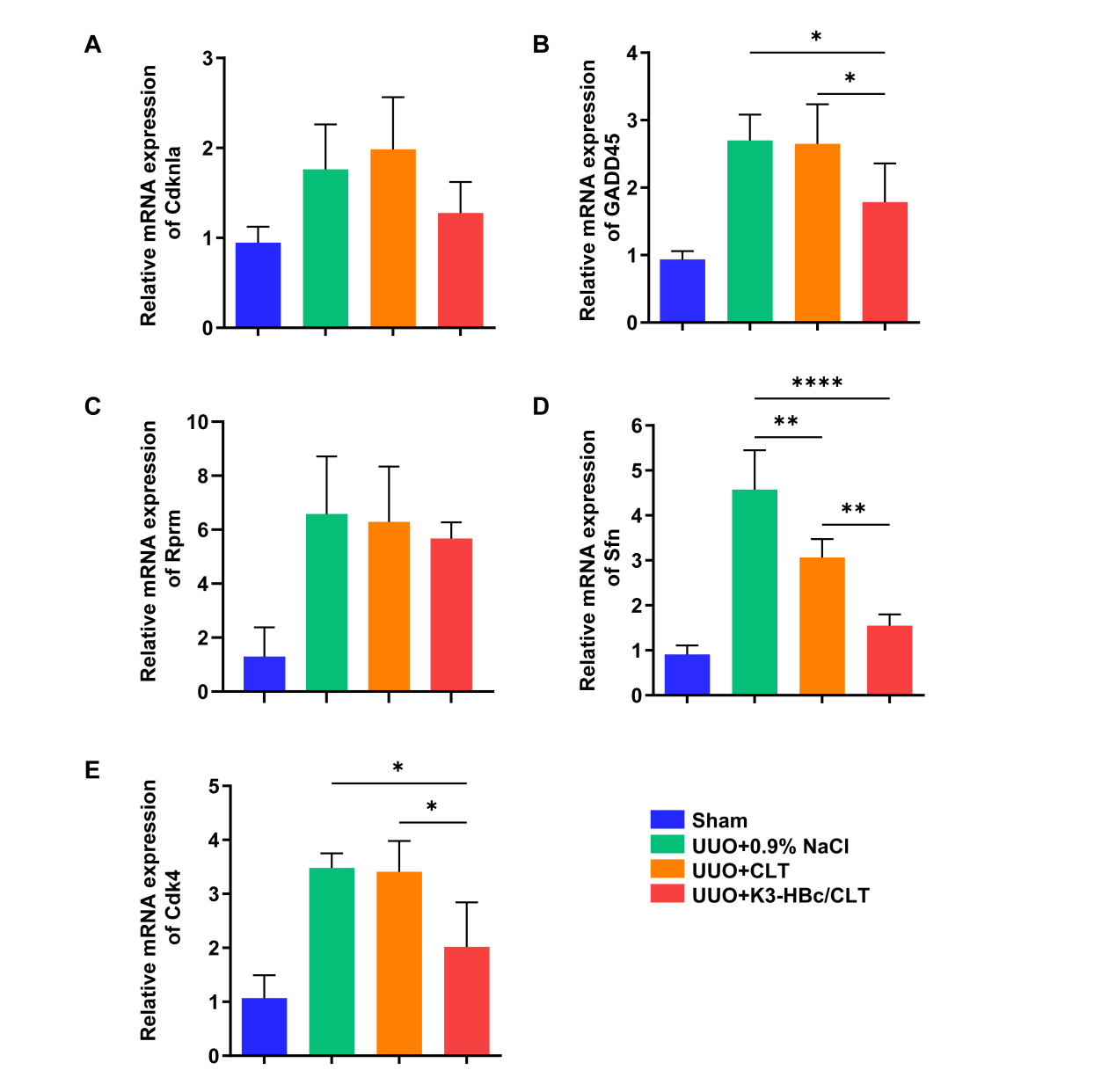


**Figure S11.** mRNA levels of (A) Cdknla, (B) GADD45, (C) Rprm, (D) Sfn, and (E) Cdk4 were measured by qPCR in obstructed kidney from the mice treated with 0.9%NaCl, CLT, or K3-HBc/CLT for 14 days (n = 3).
